# Models and challenges for studying forever chemicals and their impact on human health

**DOI:** 10.1242/dmm.052320

**Published:** 2025-10-17

**Authors:** Jana Jass, Y. Bezabhe, Majid Mustafa, Daniel Ragnvaldsson, Per-Erik Olsson

**Affiliations:** ^1^ The Life Science Center, School of Science and Technology, S-701 82 Örebro University, Sweden; ^2^ Envix Nord AB, Kuratorvägen 2B, S-907 36 Umeå, Sweden; ^3^ Department of Environmental Science, S-10691 Stockholm University, Sweden

**Keywords:** PFAS, Model systems, Effects, Mechanism of action

## Abstract

Per- and polyfluoroalkyl substances (PFAS), also known as ‘forever chemicals’, are of high concern for human and ecosystem health. PFAS were first synthesised and developed in the late 1930s, and are now commonplace in many everyday objects, such as frying pans, food packaging and cleaning products. Due to their long half-life, these chemicals remain at high concentrations in both the environment and within exposed organisms, where they have toxic effects. Several model and animal models have been developed to help determine the deleterious effects of PFAS, which has led to the identification of multiple pathways and mechanisms that are affected or presumed to be affected. In this Review, we present an overview of PFAS and discuss possible effects on humans and wildlife. We discuss the pros and cons of various vertebrate and invertebrate model systems that have been used to study PFAS. Finally, to further address these chemicals in the future, we discuss different approaches to removing PFAS from the environment.

## Teaser

**Summary:** To better understand the impact of PFAS compounds on humans and the environment it is urgently needed that they are studied at the lower concentrations that the general public and environment is exposed to. The present review tries to give an overview of models that can contribute to this.

## Introduction

Per- and polyfluoroalkyl substances (PFAS) are called ‘forever chemicals’ due to their extreme persistence, making them of high concern for human and ecosystem health. PFAS were first synthesised and developed into a potential product in the late 1930s, followed by production by 3M from 1949 (see: https://pfas.3m.com/the-science-of-fluorochemistries) (Paul et al., 2009). In 1945, polytetrafluoroethylene (PTFE), a type of PFAS, later became known and trademarked as Teflon™ (see: https://www.manufacturingdive.com/news/the-historybehind-forever-chemicals-pfas-3m-dupont-pfte-pfoa-pfos/698254/), and was used as a coating in non-stick pans due to its temperature resistance and lipophilic and hydrophobic properties (Hamid et al., 2024). Subsequently, PFAS chemicals were used widely in industrial and commercial applications, beginning in the 1950s, and are now commonplace in many products: for example, polychlorotetrafluoroethylenes (PCTFEs) are found in semiconductors and electronic components; PTFEs in medical devices, surgical implants, personal care products and non-stick cooking pans; perfluorooctanoic acid (PFOA) in waterproof clothing, electronics and building materials; and perfluorooctanesulfonic acid (PFOS) in water-repelling products and firefighting foams. Due to the vast number of PFAS chemicals and their use in numerous settings, understanding, mitigating against and eradicating their use remains a significant challenge. Yet, what knowledge do we have to guide these efforts?

Two major classes of PFAS are composed of a fluorinated alkyl chain of various length – which determines whether the compound is considered short- or long-chained – and a polar head group consisting of either sulfonic acid, i.e. perfluoro sulfonic acids (PFSAs), or carboxylic acid, i.e. perfluoro carboxylic acids (PFCAs) (Dickman and Aga, 2022). Organofluorine compounds were first detected in human blood in 1968 and tentatively identified as PFOA in 1976 (Taves, 1968a,b; Taves et al., 1976). By the 1980s, deleterious effects of PFOA begun to emerge (Olson and Andersen, 1983; Levitt and Liss, 1986). Over the years, the number of compounds defined as PFAS molecules has increased and now ranges from 4700 to 15,000 (OECD, 2021; Wang et al., 2021; Gaines et al., 2023). Of these, only a relatively small number of compounds have been investigated for their persistence and biological effects.

**Fig. 1.**
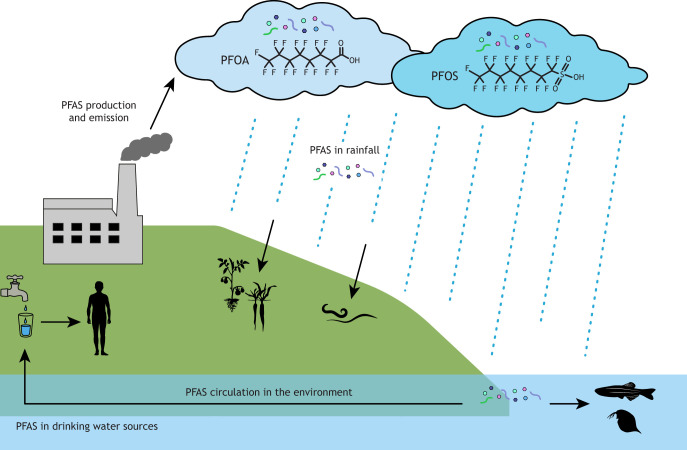
**Environmental emission of forever chemicals.** Exposure to animals and humans comes from water, food and other products emitted by humans containing PFAS that, today, are found everywhere.

PFAS chemicals are released into the environment through various sources, including from industrial practices and use in pesticides (Fig. 1). Due to their long half-life, the levels of PFAS chemicals remain high in both the environment and exposed organisms. With increasing data showing the toxicity of PFOS and PFOA, and the impending restrictions on their use, there has been a move to alternative replacement molecules (Wang et al., 2009, 2013, 2023). Although the replacement chemicals often have shorter half-lives, they are not necessarily less toxic to living organisms. Due to the ubiquitous presence of PFAS in different environments, they remain a significant threat to both environment and to human health (Fig. 1).

These health concerns have prompted the introduction of several legal restrictions and initiated the phasing out of long-chain PFAS (Box 1). This has also led to the introduction of many new variants and substitutes of PFAS with different structural modifications, such as shorter fluoroalkyl chain length or replacement of fluorine with chlorine, to increase excretion and/or degradation (Dickman and Aga, 2022).

Box 1. Regulation and restriction of PFAS chemicals Compared to many other groups of chemicals, PFAS were largely unregulated until the early 2000s (see: https://www.manufacturingdive.com/news/the-historybehind-forever-chemicals-pfas-3m-dupont-pfte-pfoa-pfos/698254/); however, bans and legislations against PFAS have been rapidly developed and introduced more recently. In 2002, the company 3M, who supplies Scotchgard products, stopped using PFOS in their products. By 2007, the US environment protection agency (USEPA) introduced new user regulations on the manufacturing and use of selected PFAS compounds (Hamid et al., 2024).

Several PFAS compounds have been designated as persistent organic pollutants (POPs) under the Stockholm Convention due to their persistent, toxic and bioaccumulative characteristics in the environment (ECHA 2024, see: https://echa.europa.eu/hot-topics/perfluoroalkyl-chemicals-pfas). Further, under the regulation of POPs by the EU (https://eur-lex.europa.eu/eli/reg/2019/1021/oj/eng), PFOS was banned in 2009 and, in July 2020, PFOA – including its salts and PFOA-derivates – were included in the POPs regulation. In addition, PFHxS, its salts and related compounds became part of the POPs regulation ban in August 2023 (ECHA 2024, see: https://echa.europa.eu/hot-topics/perfluoroalkyl-chemicals-pfas). Although Europe enforced legal restrictions on PFAS use earlier than the US, a similar regulatory development was recently implemented by the USEPA. This involved a strategic ‘road map’ on increasing knowledge of PFAS, as well as reducing, restricting and remediating PFAS through new regulations together with active clean-up activities in all relevant sectors (USEPA 2024, see: https://www.epa.gov/pfas/pfas-strategic-roadmap-epas-commitments-action-2021-2024). The most-recent update in this vast undertaking was the introduction of recommended freshwater aquatic life ambient water quality criteria (https://www.federalregister.gov/documents/2024/10/07/2024-23024/final-recommended-aquatic-life-criteria-and-benchmarks-for-select-pfas) for both PFOA and PFOS (USEPA 2024, see: https://www.epa.gov/system/files/documents/2024-09/pfoa-pfos-pfas-final-factsheet-2024.pdf).

Although many of the mechanisms of PFAS toxicity are conserved across species, significant differences exist in the extent and nature of these effects. For instance, studies have shown that fish exposed to PFAS exhibit altered endocrine function and reproductive outcomes, which may not be directly translatable to human health (Gui et al., 2023; Wasel et al., 2021). Moreover, the metabolic pathways employed by different organisms can influence the toxicity of PFAS, as seen in the varying responses of rodents and humans to these compounds (Bjork et al., 2011). Meanwhile, human epidemiological data and biomarkers suffer from confounding factors that limit mechanistic understanding of PFAS-induced effects. Therefore, controlled animal experiments with detailed biochemical analyses have been crucial in understanding mechanisms of PFAS toxicity and refining human health-risk assessments. Differences in exposure levels, toxicokinetics and tissue distribution necessitate caution when extrapolating species-specific findings to humans for accurate assessment of risk and developing effective regulatory strategies (Beale et al., 2022; Pizzurro et al., 2019; Roth et al., 2020).

In this Review, we provide an overview of PFAS and their potential adverse effects on both humans and wildlife. We also discuss model systems, both vertebrates and invertebrates, that have been used to study PFAS toxicity. In addition, we present current approaches for removing PFAS from the environment. Finally, we discuss future perspectives on how to address the challenges posed by PFAS as well as other forever chemicals.

## Bioaccumulation of PFAS chemicals

**Fig. 2.**
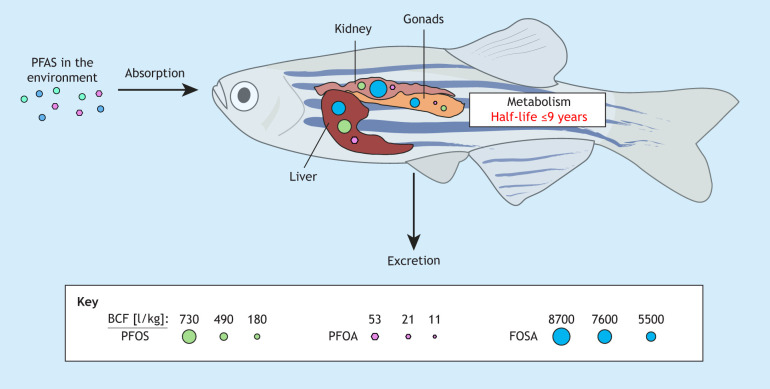
**Absorption, distribution, metabolism and excretion of PFAS compounds in Fathead Minnow.** Although the uptake and distribution of PFAS leads to high bioaccumulation factors in animals and humans, the slow metabolism and excretion lead to long half-lives of these forever chemicals. BCF; bioconcentration factor (figure adapted from Hill et al., 2024).

PFAS-polluted water is a major source of PFAS bioaccumulation in organisms. Surface and drinking waters reveal high variation in PFAS abundance, with microgram-per-litre levels observed in some countries associated with the production or use of firefighting foam, and nanogram-per-litre levels in other countries (Wee and Aris, 2023). The highest PFAS levels in drinking water sources were found in the USA (11 µg/l) and in drinking water in Sweden (8 µg/l) (Crone et al., 2019; Li et al., 2018; Gobelius et al., 2018). The bioaccumulation factor (BAF), i.e. the extent of accumulation within an organism in relation to its environment, is influenced by many factors and must be considered when analysing effects of PFAS. In one study the median logarithm-transformed (log) BAF varied between 1 and 4.4 for different PFAS, with a median log BAF of 3.55 for PFOS and 2.16 for PFOA (Burkhard, 2021). In another study, the log bioconcentration factor (BCF), i.e. the extent of accumulation within a specific tissue/organ, was ≤8700 l/kg in the kidneys of Fathead minnows (Hill et al., 2024), indicating that PFAS compounds will reach much higher levels in organs than in the environment (Fig. 2).

The metabolism of PFAS is complex and varies significantly between different organisms. In humans, PFAS are known to have long half-lives, leading to bioaccumulation in the body (Li et al., 2018). Studies have identified that certain PFAS, such as PFOA and PFOS, undergo minimal metabolic transformation, resulting in their persistence in biological systems (reviewed by Liu et al., 2023). This limited metabolism contributes to their toxicokinetics and their potential for long-term health effects (Obiako et al., 2024; Pizzurro et al., 2019). In contrast, studies using rats and fish have shown that PFOA can be metabolised into shorter chains of perfluoroalkyl acids or transformed via oxidative pathways, leading to the formation of various metabolites that may possess distinct toxicological properties (Bjork et al., 2011; Han et al., 2021). In either case, accumulation of PFAS in the body has been linked to metabolic dysregulation through their interactions with key metabolic pathways (Beale et al., 2022; India-Aldana et al., 2023). A review of omics-based studies indicated that PFAS exposure at environmentally relevant levels mostly affects the pentose phosphate (also called the hexose monophosphate shunt) pathway in different organisms (Beale et al., 2022). In addition, the resemblance of PFAS to fatty acids enables PFAS to disrupt lipid, glucose, amino acid and energy metabolism, often with pronounced effects in the liver, a primary site of PFAS accumulation (Ojo et al., 2021; Roth et al., 2020; Alijagic et al., 2024).

## Mechanism of PFAS toxicity

Following the detection of PFAS compounds in human blood (Taves, 1968a,b; Taves et al., 1976), efforts were made to understand their effects on human health. An early study regarding these findings investigated the *in vitro* effects of PFAS on human B-cells and observed that PFOA alters immunoglobulin M (IgM) gene expression and disrupted cell membranes (Lewitt and Liss, 1986, 1987). At >400 µg/l (1 µM), PFOA was later shown to activate peroxisome proliferator-activated receptor (PPAR) alpha (PPARα) in green monkey kidney COS-1 cells (Maloney and Waxman, 1999) and human COS-1 cells (Shipley et al., 2004). Meanwhile, 20-times higher concentrations were required to activate PPAR gamma (PPARγ) (Maloney and Waxman, 1999). PPARs are nuclear receptors that regulate lipid metabolism, energy balance, inflammation and cell differentiation (Feige et al., 2006). Activation of PPARs likely serves as a molecular initiating event, linking PFAS exposure to disruption of crucial cellular functions (Evans et al., 2022). This sets off a cascade of transcriptional changes that underlie many adverse health outcomes associated with PFAS exposure, including metabolic syndrome, and immunomodulation. Following initial *in vitro* studies, an epidemiological study on pregnant women confirmed that PFOA, but not PFOS, affects foetal growth and development (Fei et al., 2008). In another study PFOA was found to correlate to pre-eclampsia and birth defects, while PFOS was observed to result in pre-eclampsia and low birth weight (Stein et al., 2009). Since these first *in vitro* and epidemiological studies, subsequent reports have expanded on the harmful effects of PFAS compounds on humans.

### Nervous system and behaviour

Activation of PPARs, particularly PPARα, disrupts lipid metabolism and adipogenesis, resulting in conditions like dyslipidemia, steatosis (i.e. fat accumulation in liver cells) and fatty liver disease. PPARs are also essential for regulating lipid metabolism and cell growth (Bjork et al., 2011; Alijagic et al., 2024). As such, PFAS exposure has been associated with elevated serum cholesterol and triglycerides in humans and animals, with lipid dysregulation observed even at low concentrations in sensitive populations (India-Aldana et al., 2023). Specific lipids – particularly glycerophospholipids, sphingolipids and cholesterol – are structural components of neuronal and myelin membranes, and are involved in the formation of dynamic microdomains (lipid rafts) that organize receptors and signalling complexes essential for synaptic transmission, neurodevelopment and glial functions (Sonnino et al., 2015; Prakash et al., 2025). PFAS-mediated dysregulation of lipid homeostasis through altered PPAR signalling is, therefore, associated with detrimental neurological effects.

A recent study showed that brain development is altered in children following maternal exposure to PFAS (with blood PFAS levels in the ng/ml range), resulting in sex-specific changes in development of the white matter of the central nervous system in young children (England-Mason et al., 2025). Meanwhile, exposure of pregnant female Spraque-Dawley rats to a PFAS mixture in drinking water resulted in changes in locomotor activity, with reduction in female and increase in male offspring (Marchese et al., 2024). PFAS have also been implicated with neurodegeneration. In a study using human induced pluripotent stem-cell-derived neurons, it was observed that exposure to PFOA between 0.04 and 0.4 parts-per-billion (µg/l) results in changes in neuronal gene expression patterns of genes associated with Alzheimer's disease (Wu et al., 2024). These studies show that exposure to environmentally relevant PFAS levels can impair nervous system function and behaviour, potentially through the disruption of lipid homeostasis, neurotransmitter systems – particularly dopamine and glutamate – calcium homeostasis and expression of synaptic proteins, thereby compromising membrane fluidity and receptor function, excitotoxicity, altered synaptic transmission as well as behavioural changes, such as anxiety- and depression-like phenotypes in animal models (Brown-Leung and Cannon, 2022).

### Immune system

Human epidemiological studies have shown a relationship between PFAS exposure and altered immune function. A strong correlation was observed between increased serum PFAS levels and a reduced antibody response following vaccination in children (Grandjean et al., 2012; Stein et al., 2015; Timmermann et al., 2020), and increased susceptibility to infectious diseases (Goudarzi et al., 2017; Dalsager et al., 2021) and in *C. elegans* models (Stylianou et al., 2019; Mangu et al., 2022). Grandjean and colleagues reported reduced vaccine antibody levels in children immunised against childhood infectious diseases (diphtheria, rubella, mumps and tetanus) following exposure to maternal serum PFAS levels, some antibody concentrations falling below those that have been established as being protective (Grandjean et al., 2012, 2017). A meta-analysis of epidemiological studies on vaccination efficiency of children exposed to PFAS (based on antibody levels) indicates a clear immunosuppression by PFOA, PFOS and perfluorohexane sulfonate (PFHxS) (Crawford et al., 2023). In another study, increased serum concentrations of PFAS have been associated with higher levels of contracting the common cold in the USA (Zhang et al., 2023) and lower respiratory infections in Norway (Kvalem et al., 2020). To date, studies have been inconsistent in correlating cytokine levels and other innate immune function markers with different levels of PFAS exposure (Phelps et al., 2024). In *C. elegans*, we observed that PFAS alters the expression of immune responsive genes, specifically in response to Gram-positive bacterial infections, thereby increasing the susceptibility of the nematodes to *Staphylococcus aureus* infection (Mangu et al., 2022). Although the underlying mechanisms are not yet known, serum levels of different PFAS have been correlated to increased activation of subpopulations of natural killer cells and T helper (T_H_) cells associated with T_H_2 and/or T_H_17 and regulatory T cells (Tregs). They have also been associated with decreased CXCR3-positive cytotoxic T cells, which together may contribute to the observed immunosuppression (Tursi et al., 2024).

Although evidence linking PFAS to allergic reactions, asthma, atopic dermatitis and respiratory infections remains limited (von Holst et al., 2021), PSAS have been implicated in allergic inflammation and the development of asthma in humans by affecting T-lymphocyte function, particularly, by polarising the T_H_2 and/or T_H_1 response towards that of T_H_2 (Fairley et al., 2007). In a murine model of asthma, PFOA exposure increased serum IgE levels and caused hypersensitivity to ovalbumin, suggesting an increased response to allergens (Fairley et al., 2007). Although these studies suggest that PFAS impact immune function, there are many inconsistencies, suggesting that multiple factors influence the immune effects, such as exposure route, life stage of exposure, duration and the composition of PFAS.

### Endocrine systems and reproduction

PFAS have been linked to disruption of endocrine signalling in humans and animals, which can lead to aberrant cell differentiation and proliferation, both of which are vital for normal cellular function and development (Gundacker et al., 2022). *In vitro* studies using luciferase reporter assays have been used to study the interactions between PFAS substances and hormonal systems. Although several of these studies indicate that PFAS can affect estrogen receptors (ESRs), androgen receptor (AR), thyroid hormone receptors (THRs), transthyretin and PPARs, high PFAS concentrations are needed for activation or inhibition. Regarding ESRs and AR, PFAS levels between 10^−3^ and 10^−5^ M are needed to affect receptor activity (Behr et al., 2018; Li et al., 2020a,b; Evans et al., 2022). As both types of receptor are activated by PFAS at 10^−11^−10^−12^ M this means that a high bioconcentration is needed for PFAS to affect these receptors. For transthyretin, the situation is different, as *in vitro* studies have shown that 10^−8^ M of PFOS and PFOA result in disruption of transthyretin transport of thyroid hormone (Carlier et al., 2024). It has also been shown that PFOA inhibits THR luciferase activity at concentrations above 10^−6^ M in an *in vitro* human cell-based assay (Sprengel et al., 2021).

Another system was shown to be affected by PFAS is that of the PPAR isoforms. Analysis of interactions between PFAS and PPARα, and PFAS and PPARγ have shown that PFAS chemicals at concentrations >10^−5^ M can activate both isoforms (Evans et al., 2022). This is close to the endogenous activation that requires 10^−8^ M to activate PPARα and 10^−6^ M for PPARγ. PFAS substances also have the potential to interfere with hormone systems if body concentrations exceed the threshold for interaction. Activation of PPARs or dysregulation of their signalling due to the presence of PFOA, PFOS, perflouorbutanesulfonic acid (PFBS) and other PFAS have been implicated in the dysregulation of cell growth, potentially resulting in cancer development in humans and animals (Singh and Hsieh, 2021; Imir et al., 2021). Estrogenic and thyroid-damaging effects of PFAS have also been suggested to contribute to reduced foetal growth and birth weight (Gundacker et al., 2022).

It has been observed that exposure to PFAS results in alterations in methylation patterns, motility and sperm quality both in humans and murine models (Joensen et al., 2009; Maxwell et al., 2024; Šabović et al., 2020). Sperm motility has been associated with disruption of the plasma membrane and, as PFAS has also been shown to disrupt mitochondrial bioenergetics, this may be another contributing factor (Šabović et al., 2020; Kleszcyński et al., 2009). A study using *Daphnia magna* (water flea) observed that PFOS and PFOA exposure results in the reduction of offspring as well as a downregulation of genes involved in reproductive processes (Seyoum et al., 2020). Exposure to PFAS mixtures has also been shown to lead to oocyte apoptosis and reduced fertility in mice (Yan et al., 2025). Thus, PFAS exposure has been shown to disturb endocrine signalling, sperm and oocyte function and reproduction.

### Cell differentiation and cancer

The impact of PFAS on cell differentiation and cancer has garnered significant attention in recent years. Epidemiological studies have linked PFAS exposure to increased risks of various cancers, including kidney and testicular cancers (Pesonen and Vähäkangas, 2024; Liu et al., 2023; Bartell and Vieira, 2021; Alexander et al., 2024). Epidemiological evidence comes from both community- and occupational-based cohorts. According to the Mid-Ohio/West Virginia ‘C8’ Health Project, some 32,000 adult residents (enrolled between 2005 and 2006) who had been exposed to PFOA via contaminated drinking water experienced significantly higher risks of kidney and testicular cancers during a follow-up in 2014 (Bartell and Vieira, 2021; Liu et al., 2023). A nested case-control study within that same community (324 kidney-cancer cases and matched controls) reinforced the PFOA–kidney cancer link (Liu et al., 2023). Likewise, in an occupational cohort of 2659 long-term employees at the 3M PFOS production plant in Decatur (AL, USA) who were employed between 1961 and 2010, investigators reported an elevated incidence of kidney cancer and suggestive increases in testicular cancer (Alexander et al., 2024). These findings across distinct populations and exposure scenarios underscore a consistent association between PFAS exposure and genitourinary malignancies. The mechanisms underlying these associations are multifaceted, involving alterations in cellular signalling pathways, gene expression and epigenetic modifications (Boyd et al., 2022). Although PFAS are not directly mutagenic, they can influence tumour development through non-genotoxic mechanisms (Pesonen and Vähäkangas, 2024). PFAS-associated oxidative stress and inflammation are key contributors to cancer development in neuronal and liver cells in humans (Obiako et al., 2024; Ojo et al., 2021). The generation of reactive oxygen species (ROS) and chronic inflammation induced by PFAS can also damage DNA, leading to genomic instability, and subsequent tumorigenesis in humans and animals (Singh and Hsieh, 2021). Recent studies using animal models have shown that PFAS exposure can alter the expression of genes involved in oxidative stress response and DNA repair mechanisms, leading to carcinogenesis (Pesonen and Vähäkangas, 2024). Emerging evidence also suggests that PFAS can induce epigenetic changes, including DNA methylation and histone modifications, which may play a crucial role in altering gene expression patterns associated with cancer development in humans and animals (Kim et al., 2021). In a human lung cancer cell line, PFAS exposure can cause hypomethylation and dysregulation of cell proliferation and apoptosis pathways (Jabeen et al., 2020).

Exposure to PFAS has also been associated with accelerated epigenetic aging and alterations in locus-specific DNA methylation in firefighter individuals (Goodrich et al., 2021). Meanwhile, a biomarker cohort study with 63 school-age children (aged 7-11 years) matched on sex, age and body mass index, who had been exposed to high levels of PFAS (median 295 ng/ml and range 190-464) through contaminated drinking water (Xu et al., 2022) found associations between PFAS exposure and alterations in DNA methylation at specific genomic positions and regions. These epigenetic alterations can persist across generations, potentially explaining the long-term health effects of PFAS exposure.

### PFAS metabolism and detoxification

PFAS impact glucose metabolism through altered insulin signalling and glucose transport. Experimental studies suggest PFAS exposure induces insulin resistance and glucose intolerance, predisposing to metabolic syndrome components such as obesity, diabetes and hypertension (Chung et al., 2022; Hyötyläinen et al., 2024; Bjork et al., 2011; Roth et al., 2020; Roth and Petriello, 2022). Additionally, disruptions in pathways involving glycerophospholipids and amino acids impair energy metabolism, affecting mitochondrial efficiency and cell membrane integrity (India-Aldana et al., 2023; Alijagic et al., 2024). Changes in insulin, leptin and adipokines contribute to metabolic disorders, compounding risks for chronic diseases, like cardiovascular disease and diabetes (Roth and Petriello, 2022).

Biotransformation of PFAS in mammals mainly occurs in the liver, which detoxifies and eliminates the compounds from the body. The cytochrome P450 enzyme system is integral to metabolic processing of PFAS and other xenobiotics. Studies in mammals have shown that specific PFAS can alter the expression of phase I and II detoxification enzymes in the liver (Franco et al., 2020). PFOA exposure significantly reduces the expression of cytochrome P450 enzymes and UDP-glucuronosyltransferase (UGT) isoforms in human liver cells, potentially disrupting metabolism of endogenous and exogenous substances (Franco et al., 2020; Liu et al., 2019). Another study found no evidence of PFOA-glucuronide formation in human and rat microsomes, suggesting PFOA is not a substrate for UGTs (Kemper and Nabb, 2005). Although these findings provide insights into the effects PFOA has on biotransformation pathways, they also highlight the complexity of its interactions with metabolic enzymes and the need for further research to fully understand its toxicological implications.

## Dissecting the effects of PFAS with non-mammalian model systems

**Fig. 3.**
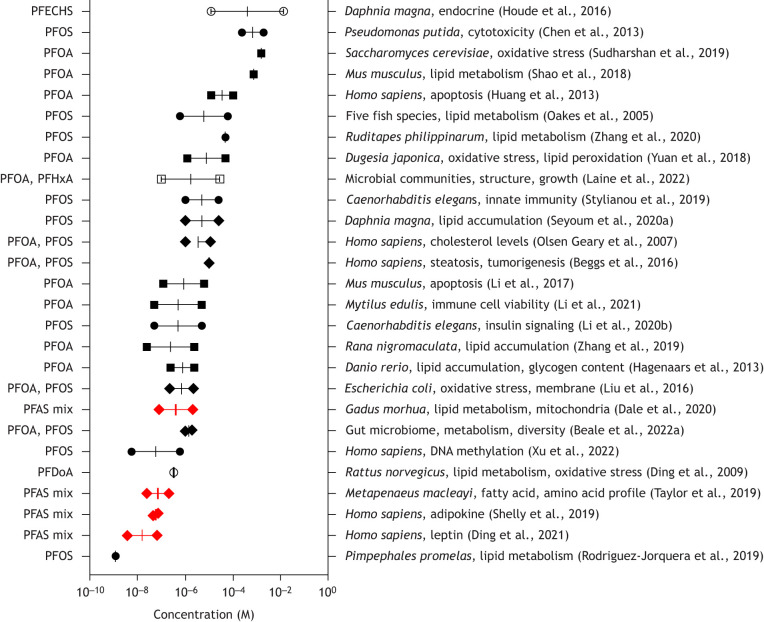
**Summary of the effects of PFAS in different organisms and at different concentrations.** The reported exposure concentration ranges (in M) are shown. Symbols represent different PFAS compounds: PFAS mixtures (◆), PFOA (■), PFOS (●), PFOA and PFOS (◆), PFOA and PFHxA (□), PFECHS (O) and PFDoA (ɸ).

In parallel to the growing evidence of health effects on humans, different model systems have been used to study the health effects of PFAS chemicals. Early studies used green monkey COS-1 cells to determine the effects of PFOA and PFOS on mouse and rat systems (Olson and Andersen, 1983; Levitt and Liss, 1986; Shipley et al., 2004). Since then, a variety of animal models have been used to study the toxicity of PFAS chemicals. Some model organisms have been selected due to their use in other research areas, in which these models had been studied in detail with their genomes sequenced, offering valuable advantages over less-studied organisms. In addition to vertebrate species, invertebrate models have proven valuable for studying PFAS bioaccumulation and toxicity. Species-specific differences and dietary habits affect the impact PFAS has on exposed herbivorous invertebrates that accumulate long-chain PFAS, mainly PFOA and PFOS and, to a lesser extent, short-chain PFAS. Carnivorous invertebrates only accumulate long-chain PFAS (Groffen et al., 2023; Rijnders et al., 2021). The use of model systems allowed the identification of different effects as well as the relative sensitivity of different responses (Fig. 3). The most-sensitive responses are associated with lipid metabolism and the function of lipids within membranes, leading to behavioural changes – as indicated by studies on zebrafish and medaka or by using organoids and molecular modelling (Hamed et al., 2024; Wang et al., 2024a,b; Lu et al., 2024; Zhao et al., 2023).

### Amphibians

In a meta-analysis of PFAS toxicity on different species, amphibians were observed to be the most-sensitive group, with a ‘no-observed-effect concentration’ (NOEC) at low µg/l concentrations (Wang et al., 2024a,b). Studies using amphibians, found that development of the northern leopard frog (*Rana pipiens*) and tiger salamander (*Ambystoma tigrinum*) is affected when these animals had been exposed to PFOA, PFOS or PFHxS at concentrations as low as 10 µg/l, although the American toad (*Anaxyrus americanus*) was less sensitive (Flynn et al., 2022). These species-specific differences in sensitivity have also been observed by others (Abercrombie et al., 2021). In a study that used exposure to short-chain PFCAs it was observed that growth of the northern leopard frog is increased when exposed to concentrations as low as 0.1 µg/l (Rohonczy et al., 2024). This sensitivity to low PFAS concentrations suggests that the northern leopard frog can be used to develop a good model system to determine PFAS toxicity (Hoover et al., 2017; Flynn et al., 2021).

### Zebrafish (*Danio rerio*)

Among the non-mammalian vertebrates, zebrafish (*Danio rerio*) stands out as a valuable model. The zebrafish was first introduced as a model studying developmental biology due to its oviparous nature, abundance of oocytes and the possibility to follow development in real time, as the oocytes are transparent.

Studies on the effects of PFOS and PFOA on zebrafish development have shown that relatively low doses (70 ng/l) result in increased mortality, yolk sac oedema and swim bladder defects (Hamed et al., 2024). Although exposure to the shorter perfluorohexanoic acid (PFHxA) resulted in more defects, including eye defects, yolk sac oedema, body axis deformities, pericardial oedema and swim bladder defects, exposure to PFHxS only resulted in swim bladder defects, showing that PFAS chemicals have severe effects on zebrafish development already at low-level exposure.

In a comparison between PFOS and two replacement PFAS, i.e. perfluoroethylcyclohexane sulphonate (PFECHS) and perfluorobutane sulphamide (FBSA), it was observed that both PFOS and FBSA led to increased abnormalities after exposure to 500 µg/l, while PFECHS responded already at 100 µg/l (Mahoney et al., 2024). Thus, the replacement PFAS were equally toxic as PFOS in this study.

By using the Japanese rice fish (*Oryzias latipes*) model system, another study observed neurological defects already at 260 ng/l exposure to PFECHS (Wang et al., 2024a,b). Measurements of swimming velocity showed deleterious effects at concentrations as low as at 80 ng/l, concordant with the effects observed in zebrafish (Hamed et al., 2024).

Other replacement PFAS, such as 6:2 chlorinated polyfluoroalkyl ether sulfonate (F-53B) and sodium *p*-perfluorous nonenoxybenzene sulfonate (OBS), have also been tested using the zebrafish model (Kalyn et al., 2023). F-53B had effects on fecundity (number of offspring produced), malformations and survival already at 5 µg/l (Shi et al., 2018). In another study, both F-53B and OBS were found to have effects on locomotion at 100 µg/l, a level comparable to that of detrimental exposure to PFOS. These studies indicate that more research is needed to test the novel PFOS replacement compounds, as they may be equally toxic as the traditional PFAS chemicals.

### Water flea (*Daphnia magna*)

*Daphnia magna*, a freshwater cladoceran (water flea), is a widely used model organism in ecotoxicology because of its sensitivity to pollutants and its ecological relevance. Transcriptomics, as well as physiological and metabolic analysis of *Daphnia magna,* revealed that PFAS impair their growth, reproduction and lifespan (Seyoum et al., 2020; Villeneuve et al., 2024). Exposure of the dry-season eggs (ephippia) of *Daphnia magna* to 10 µM PFOS or 25 µM PFOA has been shown to reduce hatching (Seyoum et al., 2020). A reduction in fecundity and increased time to the occurrence of first brood has been shown to occur with increased PFOS concentrations of >0.2 µM and >2 µM, respectively (Jeong et al., 2016). Also, PFOA concentrations of >24 µM have been shown to result in inhibition of reproduction (Yang et al., 2019). Disruption of energy metabolism and protein synthesis after exposure to PFAS have been indicated in a metabolomics study (Labine et al., 2023). A study using Oil Red O staining it has shown that both PFOS and PFOA exposure leads to lipid accumulation in *Daphnia magna* (Seyoum et al., 2020). The bioaccumulation of PFAS in *Daphnia magna* is influenced by the perfluoroalkyl chain length and hydrophobicity, with body surface sorption being a primary route of uptake (Dai et al., 2013). Collectively, the above studies demonstrate the use of *Daphnia magna* as a model for investigating PFAS toxicity and bioaccumulation, by offering insights into the molecular mechanisms of PFAS-induced effects and providing valuable data for environmental risk assessment.

### Roundworm (*Caenorhabditis elegans*)

*Caenorhabditis elegans* is small free-living bacterivorous soil nematode that is used for mechanistic toxicological studies. Because of a 60-80% gene homology between *C*. *elegans* and humans – including 65% of them corresponding to genes involved in human diseases, fully mapped cell fate, short lifespan and easy handling, they have been used to study toxic responses to PFAS (Kaletta and Hengartner, 2006). While a wide range of effects have been identified – including cytotoxicity, lethality, reproductive and developmental toxicity, metabolic disturbances, neurotoxicity, immunotoxicity and transgenerational effects – only a few studies were carried out using environmentally relevant concentrations (Ma et al., 2023). Both long-chain PFOS and PFOA have a BAF value of >1, i.e. a PFOS or PFOA concentration that is higher in the organism compared with that in the surrounding environment, with reproductive toxicity (i.e. effect on brood size) and behavioural defects (locomotion, head thrashing) observed after PFOS exposure of 0.01 µM (5 µg/l) and PFOA exposure of 0.1 µM (4 µg/l) (Chowdhury et al., 2022; Sana et al., 2021). Although short-chain PFBS and PFBA are considered less toxic than long-chain PFAS (as their BAF value is <1), they still show reproductive and neurological effects at exposure levels of >0.01 mM (3 mg/l PFBS, 2.14 mg/l PFBA) (Chowdhury et al., 2021; Sana et al., 2023). In one study, exposure to 0.1 µM PFOS and 1 µM PFOA during early life (i.e. 3-day exposure at stages from L1 larvae until mature adult) led to disruption of fatty acid and glucose metabolism, increased triglyceride synthesis and mitochondrial disfunction, suggesting an impact on obesity (Lin et al., 2022; Wei et al., 2021). Meanwhile, other studies observed that much lower levels of PFBS (25 nM) can affect resilience to heat and UV stress, and cause disruption of mitophagy by downregulation of mitophagy-related genes, including *pdr-1*, a gene related to human *PRKN*, mutation of which is related to Parkinson's disease (Shang et al., 2024). *C. elegans* also responded to accumulation of PFOS through PFOS-exposed bacterial food, demonstrating the role of trophic transfer (Stylianou et al., 2019). Although these studies show that most of the diverse health effects of PFAS occur at PFAS levels that are much higher than those encountered in the environment, *C. elegans* has proven to be an effective model for determining potential mechanisms of PFAS toxicity.

### Impacts of PFAS on model organisms: translating their relevance to human health

The detoxification pathway, particularly the regulatory Keap1−Nrf2 system, is conserved in non-mammalian species, like zebrafish, nematodes and fruit flies, although with notable variations in enzyme expression, activity and efficacy (Fuse and Kobayashi, 2017). Such conserved pathways make non-mammalian models valuable for assessing xenobiotics detoxification and understanding human diseases, but the variability in enzyme expression, activity and efficacy across species highlights the importance of understanding species-specific responses to PFAS exposure for assessing risk and designing mitigation strategies (Fuse and Kobayashi, 2017; Beale et al., 2022). As research progresses, it will become increasingly important to explore the nuances of detoxification mechanisms across different model species for a more effective approach in managing and reducing the impact of PFAS on the environment and human health. With increasing restrictions on the use of higher-order animals, the use of invertebrate model systems have become of great value as they can be used to determine the effects of PFAS at observed environmental concentrations. Each of the presented model systems have advantages when it comes to understanding the effects of PFAS chemicals on human health. These advantages are based on general mechanisms these organisms have in common with humans, as outlined in the mechanism section.

To understand the effects of PFAS compounds it is important to identify good model systems. Systems have been developed to study effects connected to both environmental risk analysis as well as extrapolation to human and other mammalian systems. Good model systems should have rapid life cycles, well-documented genetic systems and mapped physiology. The *Danio rerio*, *Daphnia magna* and *C. elegans* are all organisms that have been extensively used for these purposes, and that are well suited for toxicological studies that can be related to humans.

## Remediation and mitigation of PFAS

Alongside ongoing research efforts to better understand the impacts of PFAS on human health, there are also endeavours to remove PFAS from the environment. The United States Environmental Protection Agency (USEPA) has published a roadmap for PFAS actions that focuses on investments in research, restricted use and remediation to clean up contaminated sites and protect human health and wildlife (https://www.epa.gov/pfas/pfas-strategic-roadmap-epas-commitments-action-2021-2024). The success of remediation strategies must be monitored by biological assays, as chemical analysis of all PFAS compounds is too costly. Methods that have been tested for removing PFAS compounds from water are briefly described in the following section.

### Remediation by sorption, separation and destruction

Existing wastewater treatment plants (WWTPs) mainly focus on the removal of solids, biological pathogens, nitrogen, phosphorus and micropollutants but do not effectively remove PFAS (Appleman et al., 2014; Gobelius et al., 2023; Kim et al., 2022, 2024; Schaefer et al., 2023; Zhang et al., 2024). The fate of PFAS compounds at WWTPs is largely determined by their structures, where the carbon-chain length plays a key role (Kim et al., 2022). Long-chain (≥8C) perfluoroalkyl acids (PFAAs) are portioned with sludge, while short-chain (C3-C7) PFAAs tend to remain in aqueous phase (Kim et al., 2022). Biological treatment transforms the PFAS precursors into short-chain compounds that are only poorly removed (Kim et al., 2022).

Advanced processes, such as disinfection by chlorination and UV-sterilisation (Appleman et al., 2014; Coggan et al., 2019; Qiao et al., 2023) as well as advanced oxidation processes, have been reported to be ineffective in PFAS removal (Appleman et al., 2014; Kim et al., 2024; Qiao et al., 2023). The ineffective removal of PFAS, makes WWTPs a point source for their release into the environment.

In recent years, WWTPs have been exploring the use of granular activated carbon (GAC) as a sorption medium for PFAS removal due to its effectiveness at removing long-chain PFAS, ease of installation and low energy requirement (Appleman et al., 2014; Belkouteb et al., 2020; Inyang and Dickenson, 2017; Zhang et al., 2024). GAC is less effective at removing short-chain PFAS (Appleman et al., 2014). Organic matter substantially impacts GAC performance, as it competes with micropollutants for sorption sites and saturates the pore structure, leading to short service life of GAC (Belkouteb et al., 2020).

Another approach is membrane filtration that separates PFAS from water by size exclusion (Franke et al., 2019), electrostatic repulsion (Mabaso et al., 2024) or diffusion processes (Araújo et al., 2022). Low-pressure membranes are unable to remove PFAS because their pore size is larger than the effective diameter of PFAS molecules (Appleman et al., 2014; Rahman et al., 2014). While high-pressure membranes, including nanofiltration and reverse osmosis, can achieve >99% removal of PFAS (Appleman et al., 2014; Franke et al., 2021; McCleaf et al., 2023), the lifespan of membranes is negatively impacted on by organic matter, inorganic ions and particles (Taher et al., 2024). Another key issue is the concentrated rejected stream generated by membrane filtration (Franke et al., 2021; McCleaf et al., 2023).

Although UV treatment itself is ineffective in degrading PFAS, it can be beneficial when combined with other processes and chemicals (Dai et al., 2019; Fennell et al., 2022; Uwayezu et al., 2023). UV disinfection combined with reduction of sulfite and iodide levels of the agent, achieved >99.7% removal of most PFSAs and PFCAs, and >90% defluorination (Liu et al., 2022).

Electrochemical oxidations show a high degradation efficiency for both long- and short-chain PFAS (Mirabediny et al., 2023). Among different tested anodes, boron-doped diamond electrodes are the most efficient, resulting in >90% degradation (Lin et al., 2013). As contaminants are degraded at the anode surface, the degradation rate is limited by mass transfer (Smith et al., 2023), which is an obstacle for handling large flows. It can be combined with other processes, such as air-fractionation (Smith et al., 2023) and membrane filtration (Urtiaga, 2021), which concentrates PFAS into a significantly smaller volume. A single remediation process is unable to remove PFAS; therefore, a combination of processes in a treatment chain approach is needed.

### Bioremediation

Bioremediation is considered a more sustainable and environmentally friendly *in situ* technology for PFAS remediation. Although bioaccumulation and biotransformation of PFAS by plants, earthworms and soil microorganisms have been reported, they are currently at early stages (Gobelius et al., 2017; Beškoski et al., 2018; Zhao et al., 2020; Shahsavari et al., 2021). One study reported a decrease of 46-69% PFOS and 16-36% PFOA in supernatants after 28 days of aerobic treatment with microbial consortia established from a PFAS-contaminated site. This was attributed to adsorption to biomass, since no defluorination products had been observed (Beškoski et al., 2018). There are few reports of single bacteria, primarily *Pseudomonas* species, degrading and removing PFAS. *Pseudomonas aeruginosa* strain Hj4 removed 67-75% PFOS after 6 days but, again, no biotransformation products were observed (Kwon et al., 2014). A more-recent study using *P. aeruginosa* and *Pseudomonas putida* isolates showed respective reduction of PFOS and PFOA at 47% and 19-28% in 4 days, producing a range of short-chain perfluorinated products [PFHxA, perfluoroheptanoic acid (PFHpA), perfluoropentanoic acid (PFPeA)] (Chiriac et al., 2023). Only *Pseudomonas plecoglossicida* 2.4-D has been reported to produced PFHpA and free fluorine as transformation products with 100% efficiency after 6 days (Chetverikov et al., 2017). Other bacteria evaluated for PFAS degradation include *Acidimicrobium* sp. strain A6 (Huang and Jaffé, 2019) and *Gordonia* sp. strain NB4-1Y (Shaw et al., 2019). It remains uncertain whether the conditions required for their effective implementation can be achieved *in situ.* A more promising study involving polymeric encapsulation of a microbial consortium of *Paracoccus, Hyphomicrobium* and *Micromonosporaceae* degraded up to 74% PFOS, as evidenced by transformation products (Sorn et al., 2023). Although early results are promising, complete mineralisation has not been achieved; thus, the perfluorinated transformation products remain an issue. In addition, the challenges associated with achieving environmental conditions for effective microbial activity and their safety need to be addressed before such technology can be implemented.

Bioremediation technologies, such as mycoremediation and phytoremediation hold more promise, although they have also not yet been implemented. Mycoremediation utilizes the metabolic potential of fungi to degrade pollutants. White rot fungi are naturally able to degrade tough wood polymers due to secretion of mediators that help mobilize a diverse array of lignin- and cellulose-degrading enzymes. Filamentous white rot fungi have been shown to degrade a large range of recalcitrant organic pollutants, including PFAS (Torres-Farradá et al., 2024). Several other white rot fungi species have been shown to degrade PFAS to varying degrees, including *Pleurotus ostreatus* (PFOA, 50% in 157 days) (Luo et al., 2015), *Phanerochaete chrysosporium* [6:2 fluorotelomer alcohol (FTOH), 50% and 8:2 FTOH both in 28 days] (Tseng et al., 2014), and *Gloephyllum trabeum* and *Trametes versicolor* (6:2 FTOH into transformation products in 28 days) (Merino et al., 2018). The major challenges in implementing mycoremediation are the slow degradation rates, suitable substrate for fungal growth and the activation of the enzyme for efficient degradation. Li and co-workers (2022) recently developed a lignocellulose-based lattice structure to effectively bind PFAS and support active growth of the white rot fungus *Irpex lateus* to promote PFAS degradation. The group showed that, by increasing fungal growth and simulating enzyme production within the matrix, PFOS and PFOA are transformed into short-chain PFAS after 2 weeks (Li et al., 2022). Although complete mineralisation had not been shown, such studies hold a potential for future *in situ* bioremediation strategies.

Phytoremediation is another promising approach for stabilizing and removing PFAS from soil and ground water; however, there is no evidence of biodegradation. For a long time, plants have been used to remediate toxic substances from the environment by uptake and accumulation, and various plant species have now been shown to accumulate PFAS (Bizkarguenaga et al., 2016; Gobelius et al., 2017; Nassazzi et al., 2023). In a greenhouse study, we recently reported the accumulation of PFAS in willow and poplar, showing an overall uptake of 5.6% and 4.9% of total PFAS (i.e. C4-14 perfluoroalkyl carboxylates and C4-8 perfluoroalkyl sulfonates), respectively, after 90 days (Nassazzi et al., 2025). The uptake was related to biomass with longer C-F chain-length remaining in the roots, while the short-chain PFAS moved to the biomass above ground. A recent review of phytoremediation of PFAS in soil and water indicated that this is a promising cost-effective approach for *in situ* remediation (Kavusi et al., 2023).

## Conclusions and outlook

The use of diverse model systems contributes to our understanding of PFAS toxicity mechanisms and potential adverse health outcomes in humans and ecosystems. Selection of specific model organisms for assessing PFAS accumulation and environmental impact depends on bioavailability and accumulation, and dietary habits of the organism. As research continues, these models will provide further insights into the mechanisms of PFAS toxicity and contribute to strategies for mitigating their deleterious effects. From these studies it is apparent that greater focus must be on environmentally relevant exposures in order to obtain a better and more accurate understanding of effects that can be expected. Although there are some hotspots of exposure at µg-mg/l concentrations, the general exposure levels are in the lower ng/l range. As these substances are not easily metabolised, they can accumulate up to 10,000 times higher concentrations in organs of exposed organisms. Thus, when performing *in vitro* studies, it may be that µg/l concentrations are justified. In addition, when more data on the toxicity of individual PFAS substances become available, the development of a ranking system in toxicity and environmental hazard would be valuable for improved risk assessment. Such ranking system should be based on absolute toxicity measure (e.g. species sensitivity distribution data and derivation of NOEC or HC5 values) but should also consider bioaccumulative properties and half-life expectancies during ambient conditions for different PFAS compounds to more closely relate to the accumulated levels.

PFAS chemicals have a high similarity to fatty acids and the most-sensitive effects occur when lipid metabolism is affected, leading to behavioural disturbances. As our understanding of the relationship between PFAS exposure and deleterious effects continues to evolve, a need to elucidate the full spectrum of biological effects these substances initiate is needed. In addition, whether adaptation mechanisms are in place following low-level exposure is also relevant to address. This includes a deeper investigation into the specific pathways affected by PFAS and the potential for developing strategies to mitigate these risks for individuals exposed to PFAS. The implications of this research stretch far beyond academic inquiry, potentially informing public health policies and regulatory measures aimed at reducing exposure to these harmful substances.

## References

[DMM052320C1] Abercrombie, S. A., de Perre, C., Iacchetta, M., Flynn, R. W., Sepúlveda, M. S., Lee, L. S. and Hoverman, J. T. (2021). Sublethal effects of dermal exposure to poly and perﬂuoroalkyl substances on postmetamorphic amphibians. *Environ. Toxicol. Chem.* 40, 717-726. 10.1002/etc.4711 32164037

[DMM052320C2] Alexander, B. H., Ryan, A., Church, T. R., Kim, H., Olsen, G. W. and Logan, P. W. (2024). Mortality and cancer incidence in perfluorooctanesulfonyl fluoride production workers. *Am. J. Ind. Med.* 67, 321-333. 10.1002/ajim.23568 38345456

[DMM052320C3] Alijagic, A., Sinisalu, L., Duberg, D., Kotlyar, O., Scherbak, N., Engwall, M., Orešič, M. and Hyötyläinen, T. (2024). Metabolic and phenotypic changes induced by PFAS exposure in two human hepatocyte cell models. *Environ. Int.* 190, 108820. 10.1016/j.envint.2024.108820 38906088

[DMM052320C4] Appleman, T. D., Higgins, C. P., Quiñones, O., Vanderford, B. J., Kolstad, C., Zeigler-Holady, J. C. and Dickenson, E. R. V. (2014). Treatment of poly- and perfluoroalkyl substances in U.S. full-scale water treatment systems. *Water Res.* 51, 246-255. 10.1016/j.watres.2013.10.067 24275109

[DMM052320C5] Araújo, R. G., Rodríguez-Hernandéz, J. A., González-González, R. B., Macias-Garbett, R., Martínez-Ruiz, M., Reyes-Pardo, H., Hernández Martínez, S. A., Parra-Arroyo, L., Melchor-Martínez, E. M., Sosa-Hernández, J. E. et al. (2022). Detection and tertiary treatment technologies of poly-and perfluoroalkyl substances in wastewater treatment plants. *Front. Environ. Sci.* 10, 864894. 10.3389/fenvs.2022.864894

[DMM052320C6] Bartell, S. M. and Vieira, V. M. (2021). Critical review on PFOA, kidney cancer, and testicular cancer. *J. Air Waste Manag. Assoc.* 71, 663-679. 10.1080/10962247.2021.1909668 33780327

[DMM052320C7] Beale, D. J., Sinclair, G. M., Shah, R., Paten, A. M., Kumar, A., Long, S. M., Vardy, S. and Jones, O. A. H. (2022). A review of omics-based PFAS exposure studies reveals common biochemical response pathways. *Sci. Environ.* 845, 157255. 10.1016/j.scitotenv.2022.157255 35817100

[DMM052320C8] Beggs, K. M., McGreal, S. R., McCarthy, A., Gunewardena, S., Lampe, J. N., Lau, C. and Apte, U. (2016). The role of hepatocyte nuclear factor 4-alpha in perfluorooctanoic acid- and perfluorooctanesulfonic acid-induced hepatocellular dysfunction. *Toxicol. Appl. Pharmacol.* 304, 18-29. 10.1016/j.taap.2016.05.001 27153767 PMC5367386

[DMM052320C9] Behr, A. C., Lichtenstein, D., Braeuning, A., Lampen, A. and Buhrke, T. (2018). Perfluoroalkylated substances (PFAS) affect neither estrogen and androgen receptor activity nor steroidogenesis in human cells in vitro. *Toxicol. Lett.* 291, 51-60. 10.1016/j.toxlet.2018.03.029 29601859

[DMM052320C11] Belkouteb, N., Franke, V., McCleaf, P., Köhler, S. and Ahrens, L. (2020). Removal of per- and polyfluoroalkyl substances (PFASs) in a full-scale drinking water treatment plant: long-term performance of granular activated carbon (GAC) and influence of flow-rate. *Water Res.* 182, 115913. 10.1016/j.watres.2020.115913 32585466

[DMM052320C12] Beškoski, V. P., Yamamoto, A., Nakano, T., Yamamoto, K., Matsumura, C., Motegi, M., Beškoski, L. S. and Inui, H. (2018). Defluorination of perfluoroalkyl acids is followed by production of monofluorinated fatty acids. *Sci. Total Environ.* 636, 355-359. 10.1016/j.scitotenv.2018.04.243 29709852

[DMM052320C13] Bizkarguenaga, E., Zabaleta, I., Mijangos, L., Iparraguirre, A., Fern Andez, L., Prieto, A. and Zuloaga, O. (2016). Uptake of perfluorooctanoic acid, perfluorooctane sulfonate and perfluorooctane sulfonamide by carrot and lettuce from compost amende soil. *Sci. Total Environ.* 571, 444-451. 10.1016/j.scitotenv.2016.07.010 27450950

[DMM052320C14] Bjork, J. A., Butenhoff, J. L. and Wallace, K. B. (2011). Multiplicity of nuclear receptor activation by PFOA and PFOS in primary human and rodent hepatocytes. *Toxicology* 288, 8-17. 10.1016/j.tox.2011.06.012 21723365

[DMM052320C15] Boyd, R. I., Ahmad, S., Singh, R., Fazal, Z., Prins, G. S., Madak Erdogan, Z., Irudayaraj, J. and Spinella, M. J. (2022). Toward a mechanistic understanding of poly- and perfluoroalkylated substances and cancer. *Cancers* 14, 2919. 10.3390/cancers14122919 35740585 PMC9220899

[DMM052320C16] Brown-Leung, J. M. and Cannon, J. R. (2022). Neurotransmission targets of per- and polyfluoroalkyl substance neurotoxicity: mechanisms and potential implications for adverse neurological outcomes. *Chem. Res. Toxicol.* 35, 1312-1333. 10.1021/acs.chemrestox.2c00072 35921496 PMC10446502

[DMM052320C17] Burkhard, L. P. (2021). Evaluation of published bioconcentration factor (BCF) and bioaccumulation Factor (BAF) data for per- and polyfluoroalkyl substances across aquatic species. *Environ. Toxicol. Chem.* 40, 1530-1543. 10.1002/etc.5010 33605484

[DMM052320C18] Carlier, M. P., Cenijn, P. H., Baygildiev, T., Irwan, J., Escher, S. E., van Duursen, M. B. M. and Hamers, T. (2024). Profiling the endocrine-disrupting properties of triazines, triazoles and shoer-chain PFAS. *Toxicol. Sci.* 202, 250-264. 10.1093/toxsci/kfae131 39365753 PMC11589101

[DMM052320C19] Chen, H., Yao, J., Wang, F. and Cai, M. and Liu H. (2013). Toxicity of perfluorooctanoic acid to Pseudomonas putida in the aquatic environment. *J. Hazard. Mater.* 262, 726-731. 10.1016/j.jhazmat.2013.09.046 24140521

[DMM052320C20] Chetverikov, S. P., Sharipov, D. A., Korshunova, T. Y. and Loginov, O. N. (2017). Degradation of perfluorooctanyl sulfonate by strain Pseudomonas plecoglossicida2.4-D. *Appl. Biochem. Microbiol.* 53, 533-538. 10.1134/S0003683817050027

[DMM052320C21] Chiriac, F. L., Stoica, C., Iftode, C., Pirvu, F., Petre, V. A., Paun, I., Pascu, L. F., Vasile, G. G. and Nita-Lazar, M. (2023). Bacterial biodegradation of perfluorooctanoic acid (PFOA) and Perfluorosulfonic Acid (PFOS) using pure *Pseudomonas* Strains. *Sustainability* 15, 14000. 10.3390/su151814000

[DMM052320C22] Chowdhury, M. I., Sana, T., Panneerselvan, L., Dharmarajan, R. and Megharaja, M. (2021). Acute toxicity and transgenerational effects of perfluorobutane sulfonate on *Caenorhabditis elegans*. *Environ. Toxicol. Chem.* 40, 1971-1980. 10.1002/etc.5055 33792982

[DMM052320C23] Chowdhury, M. I., Sana, T., Panneerselvan, L., Sivaram, A. K. and Megharaja, M. (2022). Perfluorooctane sulfonate (PFOS) induces several behavioural defects in *Caenorhabditis elegans* that can also be transferred to the next generations. *Chemosphere* 291, 132896. 10.1016/j.chemosphere.2021.132896 34780740

[DMM052320C24] Chung, S. M., Heo, D.-G., Kim, J.-H., Yoon, J. S., Lee, H. W., Kim, J.-Y., Moon, J. S. and Won, K. C. (2022). Perfluorinated compounds in adults and their association with fasting glucose and incident diabetes: a prospective cohort study. *Environ. Health.* 21, 101. 10.1186/s12940-022-00915-2 36289510 PMC9597959

[DMM052320C25] Coggan, T. L., Moodie, D., Kolobaric, A., Szabo, D., Shimeta, J., Crosbie, N. D., Lee, E., Fernandes, M. and Clarke, B. O. (2019). An investigation into per- and polyfluoroalkyl substances (PFAS) in nineteen Australian wastewater treatment plants (WWTPs). *Heliyon* 5, e02316. 10.1016/j.heliyon.2019.e02316 31485522 PMC6716228

[DMM052320C26] Crawford, L., Halperin, S. A., Dzierlenga, M. W., Skidmore, B., Linakis, M. W., Nakagawa, S. and Longnecker, M. P. (2023). Systematic review and meta-analysis of epidemiologic data on vaccine response in relation to exposure to five principal perfluoroalkyl substances. *Environ. Int.* 172, 107734. 10.1016/j.envint.2023.107734 36764183

[DMM052320C27] Crone, B. C., Speth, T. F., Wahman, D. G., Smith, S. J., Abulikemu, G., Kleiner, E. J. and Pressman, J. G. (2019). Occurrence of per- and polyfluoroalkyl substances (PFAS) in source water and their treatment in drinking water. *Crit. Rev. Environ. Sci. Technol.* 49, 2359-2396. 10.1080/10643389.2019.1614848 32831535 PMC7433796

[DMM052320C28] Dai, Z., Xia, X., Guo, J. and Jiang, X. (2013). Bioaccumulation and uptake routes of perfluoroalkyl acids in Daphnia magna. *Chemosphere* 90, 1589-1596. 10.1016/j.chemosphere.2012.08.026 22967930

[DMM052320C29] Dai, X., Xie, Z., Dorian, B., Gray, S. and Zhang, J. (2019). Comparative study of PFAS treatment by UV, UV/ozone, and fractionations with air and ozonated air. *Environ. Sci.* 5, 1897-1907.

[DMM052320C30] Dale, K., Yadetie, F., Müller, M. B., Pampanin, D. M., Gilabert, A., Zhang, X., Tairova, Z., Haarr, A., Lille-Langøy, R. and Lyche, J. L. et al. (2020). Proteomics and lipidomics analyses reveal modulation of lipid metabolism by perfluoroalkyl substances in liver of Atlantic cod (Gadus morhua). *Aquat. Toxicol.* 227, 105590. 10.1016/j.aquatox.2020.105590 32891021

[DMM052320C31] Dalsager, L., Christensen, N., Halekoh, U., Timmermann, C. A. G., Nielsen, F., Kyhl, H. B., Husby, S., Grandjean, P., Jensen, T. K. and Andersen, H. R. (2021). Exposure to perfluoroalkyl substances during fetal life and hospitalization for infectious disease in childhood: a study among 1,503 children from the Odense Child Cohort. *Environ. Int.* 149, 106395. 10.1016/j.envint.2021.106395 33508532

[DMM052320C32] Dickman, R. A. and Aga, D. S. (2022). A review of recent studies on toxicity, sequestration, and degradation of per- and polyfluoroalkyl substances (PFAS). *J. Hazard. Mater.* 436, 129120. 10.1016/j.jhazmat.2022.129120 35643010 PMC12981655

[DMM052320C33] Ding, L., Hao, F., Shi, Z., Wang, Y., Zhang, H., Tang, H. and Dai, J. (2009). Systems biological responses to chronic perfluorododecanoic acid exposure by integrated metabonomic and transcriptomic studies. *J. Proteom. Res.* 8, 2882-2891. 10.1021/pr9000256 19378957

[DMM052320C34] Ding, N., Karvonen-Gutierrez, C. A., Herman, W. H., Calafat, A. M., Mukherjee, B. and Park, S. K. (2021). Associations of perfluoroalkyl and polyfluoroalkyl substances (PFAS) and PFAS mixtures with adipokines in midlife women. *Int. J. Hyg. Environ. Health* 235, 113777. 10.1016/j.ijheh.2021.113777 34090141 PMC8207532

[DMM052320C35] England-Mason, G., Reardon, A. J. F., Reynolds, J. E., Grohs, M. N., MacDonlad, A. M., Kinniburgh, D. W., Martin, J. W., Lbel, C. and Dewey, D. (2025). Maternal concentrations of perfluoroalkyl sulfonates and alterations in white matter miscrostructure in the developing brians of young children. *Environ. Res.* 267, 120638. 10.1016/j.envres.2024.120638 39681179

[DMM052320C36] Evans, N., Conley, J. M., Cardon, M., Hartig, P., Medlock-Kakaley, E. and Earl Gray, L., Jr. (2022). In vitro activity of a panel of per- and polyfluoroalkyl substances (PFAS), fatty acids, and pharmaceuticals in peroxisome proliferator-activated receptor (PPAR) alpha, PPAR gamma and estrogen receptor assays. *Toxicol. Appl. Pharmacol.* 449, 116136. 10.1016/j.taap.2022.116136 35752307 PMC9341220

[DMM052320C37] Fairley, K. J., Purdy, R., Kearns, S., Anderson, S. E. and Meade, B. J. (2007). Exposure to the immunosuppressant, perfluorooctanoic acid, enhances the murine IgE and airway hyperreactivity response to ovalbumin. *Toxicol. Sci.* 97, 375-383. 10.1093/toxsci/kfm053 17369199

[DMM052320C38] Fei, C., McLaughlin, J. K., Tarone, R. E. and Olsen, J. (2008). Fetal growth indicators and perfluorinated chemicals: a study in the Danish National Birth Cohort. *Am. J. Epidemiol.* 167, 66-72. 10.1093/aje/kwn095 18460444

[DMM052320C39] Feige, J. N., Gelman, L., Michalik, L., Desvergne, B. and Wahli, W. (2006). From molecular action to physiological outputs: peroxisome proliferator-activated receptors are nuclear receptors at the crossroads of key cellular functions. *Prog. Lipid Res.* 45, 120-159. 10.1016/j.plipres.2005.12.002 16476485

[DMM052320C40] Fennell, B. D., Mezyk, S. P. and McKay, G. (2022). Critical review of UV-advanced reduction processes for the treatment of chemical contaminants in water. *ACS Environ. Au.* 2, 178-205. 10.1021/acsenvironau.1c00042 37102145 PMC10114900

[DMM052320C41] Flynn, R. W., Iacchetta, M., de Perre, C., Lee, L., Sepulveda, M. S. and Hoverman, J. T. (2021). Chronic per-/polyfluoroalkyl substance exposure under environmentally relevant conditions delays development in northern leopard frog (Rana pipiens) larvae. *Environ. Toxicol. Chem.* 40, 711-716. 10.1002/etc.4690 32072676

[DMM052320C42] Flynn, R. W., Hoover, G., Iacchetta, M., Guffey, S., de Perre, C., Huerta, B., Li, W., Hoverman, J. T., Lee, L. and Sepulveda, M. S. (2022). Comparative toxicity of aquatic per- and polyfluoroalkyl substance exposure in three species of amphibians. *Environ. Toxicol. Chem.* 41, 1407-1415. 10.1002/etc.5319 35199880 PMC9314107

[DMM052320C43] Franco, M. E., Sutherland, G. E., Fernandez-Luna, M. T. and Lavado, R. (2020). Altered expression and activity of phase I and II biotransformation enzymes in human liver cells by perfluorooctanoate (PFOA) and perfluorooctane sulfonate (PFOS). *Toxicology* 430, 152339. 10.1016/j.tox.2019.152339 31809754

[DMM052320C44] Franke, V., McCleaf, P., Lindegren, K. and Ahrens, L. (2019). Efficient removal of per- And polyfluoroalkyl substances (PFASs) in drinking water treatment: Nanofiltration combined with active carbon or anion exchange. *Environ. Sci.* 5, 1836-1843.

[DMM052320C45] Franke, V., Ullberg, M., McCleaf, P., Wålinder, M., Köhler, S. J. and Ahrens, L. (2021). The price of really clean water: combining nanofiltration with granular activated carbon and anion exchange resins for the removal of per- and polyfluoralkyl substances (PFASs) in drinking water production. *ACS ES&T Water* 1, 782-795. 10.1021/acsestwater.0c00141

[DMM052320C46] Fuse, Y. and Kobayashi, M. (2017). Conservation of the Keap1-Nrf2 system: an evolutionary journey through stressful space and time. *Molecules* 22, 436. 10.3390/molecules22030436 28282941 PMC6155405

[DMM052320C47] Gaines, L. G. T., Sinclair, G. and Williams, A. J. (2023). A proposed approach to defining per- and polyfluoroalkyl substances (PFAS) based on molecular structure and formula. *Integr. Environ. Assess. Manag.* 19, 1333-1337. 10.1002/ieam.4735 36628931 PMC10827356

[DMM052320C49] Gobelius, L., Lewis, J. and Ahrens, L. (2017). Plant uptake of per- and polyfluoroalkyl substances at a contaminated fire training facility to evaluate the phytoremediation potential of various plant species. *Environ. Sci. Technol.* 51, 12602-12610. 10.1021/acs.est.7b02926 28972370

[DMM052320C48] Gobelius, L., Hedlund, J., During, W., Tröger, R., Lilja, K. and Wiberg, K. (2018). Per- and polyfluoroalkyl substances in Swedish groundwater and surface water: implications for environmental quality standards and drinking water guidelines. *Environ. Sci. Technol.* 52, 4340-4349. 10.1021/acs.est.7b05718 29527894

[DMM052320C50] Gobelius, L., Glimstedt, L., Olsson, J., Wiberg, K. and Ahrens, L. (2023). Mass flow of per- and polyfluoroalkyl substances (PFAS) in a Swedish municipal wastewater network and wastewater treatment plant. *Chemosphere* 336, 139182. 10.1016/j.chemosphere.2023.139182 37302497

[DMM052320C51] Goodrich, J. M., Calkins, M. M., Caban-Martinez, A. J., Stueckle, T., Grant, C., Calafat, A. M., Nematollahi, A., Jung, A. M., Graber, J. M., Jenkins, T. et al. (2021). Per- and polyfluoroalkyl substances, epigenetic age and DNA methylation: a cross-sectional study of firefighters. *Epigenomics* 13, 1619-1636. 10.2217/epi-2021-0225 34670402 PMC8549684

[DMM052320C52] Goudarzi, H., Miyashita, C., Okada, E., Kashino, I., Chen, C. J., Ito, S., Araki, A., Kobayashi, S., Matsuura, H. and Kishi, R. (2017). Prenatal exposure to perfluoroalkyl acids and prevalence of infectious diseases up to 4years of age. *Environ. Int.* 104, 132-138. 10.1016/j.envint.2017.01.024 28392064

[DMM052320C53] Grandjean, P., Heilmann, C., Weihe, P., Nielsen, F., Mogensen, U. B. and Budtz-Jorgensen, E. (2017). Serum vaccine antibody concentrations in adolescents exposed to perfluorinated compounds. *Environ. Health Perspect.* 125, 077018. 10.1289/EHP275 28749778 PMC5744724

[DMM052320C54] Grandjean, P., Andersen, E. W., Budtz-Jorgensen, E., Nielsen, F., Molbak, K., Weihe, P. and Heilmann, C. (2012). Serum vaccine antibody concentrations in children exposed to perfluorinated compounds. *JAMA* 307, 391-397. 10.1001/jama.2011.2034 22274686 PMC4402650

[DMM052320C55] Groffen, T., Prinsen, E., Devos Stoffels, O.-A., Maas, L., Vincke, P., Lasters, R., Eens, M., Bervoets, L., et al. (2023). PFAS accumulation in several terrestrial plant and invertebrate species reveals species-specific differences. *Environ. Sci. Pollut. Res.* 30, 23820-23835. 10.1007/s11356-022-23799-8 36331738

[DMM052320C56] Gui, W., Guo, H., Wang, C., Li, M., Jin, Y., Zhang, K., Dai, J. and Zhao, Y. (2023). Comparative developmental toxicities of zebrafish towards structurally diverse per- and polyfluoroalkyl substances. *Sci. Tot. Environ.* 902, 166569. 10.1016/j.scitotenv.2023.166569 37633367

[DMM052320C57] Gundacker, C., Audouze, K., Widhalm, R., Granitzer, S., Forsthuber, M., Jornod, F., Wieloe, M., Long, M., Halldorsson, T. I., Uhl, M. et al. (2022). Reduced birth weight and exposure to per- and polyfluoroalkyl substances: a review of possible underlying mechanisms using the AOP-HelpFinder. *Toxics* 10, 684. 10.3390/toxics10110684 36422892 PMC9699222

[DMM052320C58] Hamed, M., Vats, A., Lim, I. E., Sapkota, B. and Abdelmoneim, A. (2024). Effects of developmental exposure to individual and combined PFAS on development and behavioral stress responses in larval zebrafish. *Environ. Pollut.* 15, 123912. 10.1016/j.envpol.2024.123912 38570156

[DMM052320C59] Hamid, N., Junaid, M., Sultan, M., Yoganandham, S. T. and Chuan, O. M. (2024). The untold story of PFAS alternatives: Insights into the occurrence, ecotoxicological impacts, and removal strategies in the aquatic environment. *Water Res.* 250, 121044. 10.1016/j.watres.2023.121044 38154338

[DMM052320C60] Han, J., Gu, W., Barrett, H., Yang, D., Tang, S., Sun, J., Liu, J., Krause, H. M., Houck, K. A. and Peng, H. (2021). A roadmap to the structure-related metabolism pathways of per- and polyfluoroalkyl substances in the early life stages of zebrafish (*Danio rerio*). *Environ. Health Perspect.* 129, 77004. 10.1289/EHP7169 34288731 PMC8294803

[DMM052320C61] Hagenaars, A., Vergauwen, L., Benoot, D., Laukens, K. and Knapen, D. (2013). Mechanistic toxicity study of perfluorooctanoic acid in zebrafish suggests mitochondrial dysfunction to play a key role in PFOA toxicity. *Chemosphere* 91, 844-856. 10.1016/j.chemosphere.2013.01.056 23427857

[DMM052320C62] Hill, N. I., Becanova, J., Vojta, S., Barber, L. B., LeBlanc, D. R., Vajda, A. M., Pickard, H. M. and Lohmann, R. (2024). Bioconcentration of per- and polyfluoroalkyl substances and precursors in fathead minnow tissues environmentally exposed to aqueous film-forming foam-contaminated waters. *Environ. Toxicol. Chem.* 43, 1795-1806. 10.1002/etc.5926 38896102 PMC11552075

[DMM052320C63] Hoover, G. M., Chislock, M. F., Tornabene, B. J., Guffey, S. C., Choi, Y. J., de Perre, C., Hoverman, J. T., Lee, L. S. and Sepulveda, M. S. (2017). Uptake and depuration of four per/polyfluoroalkyl substances (PFASS) in northern leopard frog rana pipiens tadpoles. *Environ. Sci. Technol. Lett.* 4, 399-403. 10.1021/acs.estlett.7b00339

[DMM052320C64] Houde, M., Douville, M., Giraudo, M., Jean, K., Lépine, M., Spencer, C. and De Silva, A. O. (2016). Endocrine-disruption potential of perfluoroethylcyclohexane sulfonate (PFECHS) in chronically exposed Daphnia magna. *Environ. Pollut.* 218, 950-956. 10.1016/j.envpol.2016.08.043 27554979

[DMM052320C65] Huang, S. and Jaffé, P. R. (2019). Defluorination of perfluorooctanoic acid (PFOA) and perfluorooctane sulfonate (PFOS) by acidimicrobium sp. Strain A6. *Environ. Sci. Technol.* 53, 11410-11419. 10.1021/acs.est.9b04047 31529965

[DMM052320C66] Huang, Q., Zhang, J., Martin, F. L., Peng, S., Tian, M., Mu, X. and Shen, H. (2013). Perfluorooctanoic acid induces apoptosis through the p53-dependent mitochondrial pathway in human hepatic cells: a proteomic study. *Toxicol. Lett.* 223, 211-220. 10.1016/j.toxlet.2013.09.002 24035753

[DMM052320C67] Hyötyläinen, T., McGlinchey, A., Salihovic, S., Schubert, A., Douglas, A., Hay, D. C., O'Shaughnessy, P. J., Iredal, J. P., Shaw, S., Fowler, P. A. et al. (2024). In utero exposures to perfluoroalkyl substances and the human fetal liver metabolome in Scotland: a cross-sectional study. *Lancet Planet. Health* 8, e5-e17. 10.1016/S2542-5196(23)00257-7 38199723

[DMM052320C68] Imir, O. B., Kaminsky, A. Z., Zuo, Q. Y., Liu, Y. J., Singh, R., Spinella, M. J., Irudayaraj, J., Hu, W. Y., Prins, G. S. and Erdogan, Z. M. (2021). Per- and polyfluoroalkyl substance exposure combined with high-fat diet supports prostate cancer progression. *Nutrients* 13, 3902. 10.3390/nu13113902 34836157 PMC8623692

[DMM052320C69] India-Aldana, S., Yao, M., Midya, V., Colicino, E., Chatzi, L., Chu, J., Gennings, C., Jones, D. P., Loos, R. J., Setiawan, V. W. et al. (2023). PFAS Exposures and the human metabolome: a systematic review of epidemiological studies. *Curr. Pollut. Rep.* 9, 510-568. 10.1007/s40726-023-00269-4 37753190 PMC10520990

[DMM052320C70] Inyang, M. and Dickenson, E. R. V. (2017). The use of carbon adsorbents for the removal of perfluoroalkyl acids from potable reuse systems. *Chemosphere* 184, 168-175. 10.1016/j.chemosphere.2017.05.161 28586657

[DMM052320C71] Jabeen, M., Fayyaz, M. and Irudayaraj, J. (2020). Epigenetic modifications, and alterations in cell cycle and apoptosis pathway in A549 lung carcinoma cell line upon exposure to perfluoroalkyl substances. *Toxics* 8, 112. 10.3390/toxics8040112 33238432 PMC7711517

[DMM052320C187] Jeong, T.Y., Yuk, M.S. and Kim, S.D. (2016). Multigenerational effect of perfluorooctane sulfaonate (PFOS) on the individual fitness and population growth of Daphnia magna. *Sci. Total Environ.* 569-570, 1553-1560. 10.1016/j.scitotenv.2016.06.249 27396314

[DMM052320C72] Joensen, U. N., Bossi, R., Leffers, H., Jensen, A. A., Skakkebaek, N. E. and Jørgensen, N. (2009). Do perfluoroalkyl compounds impair human semen quality? *Environ. Health Perspect.* 117, 923-927. 10.1289/ehp.0800517 19590684 PMC2702407

[DMM052320C73] Kaletta, T. and Hengartner, M. (2006). Finding function in novel targets*: C. elegans* as a model organism. *Nat. Rev. Drug Discov.* 5, 387-399. 10.1038/nrd2031 16672925

[DMM052320C74] Kalyn, M., Lee, H., Curry, J., Tu, W., Ekker, M. and Mennigen, J. A. (2023). Effects of PFOS, F-53B and OBS on locomotor behaviour, the dopaminergic system and mitochondrial function in developing zebrafish (Danio rerio). *Environ. Pollut.* 326, 121479. 10.1016/j.envpol.2023.121479 36958660

[DMM052320C75] Kavusi, E., Ansar, B. S. K., Ebrahimi, S., Sharma, R., Ghoreishi, S. S., Nobaharan, K., Abdoli, S., Dehghanian, Z., Lajayer, B. A., Senapathi, V. et al. (2023). Critical review on phytoremediation of polyfluoroalkyl substances from environmental matrices: Need for global concern. *Environ. Res.* 217, 114844. 10.1016/j.envres.2022.114844 36403653

[DMM052320C76] Kemper, R. A. and Nabb, D. L. (2005). In vitro studies in microsomes from rat and human liver, kidney, and intestine suggest that perfluorooctanoic acid is not a substrate for microsomal UDP-glucuronosyltransferases. *Drug. Chem. Toxicol.* 28, 281-287. 10.1081/DCT-200064468 16051554

[DMM052320C77] Kim, S., Thapar, I. and Brooks, B. W. (2021). Epigenetic changes by per- and polyfluoroalkyl substances (PFAS). *Environ. Pollut.* 279, 116929. 10.1016/j.envpol.2021.116929 33751946

[DMM052320C78] Kim, J., Xin, X., Mamo, B. T., Hawkins, G. L., Li, K., Chen, Y., Huang, Q. and Huang, C. H. (2022). Occurrence and fate of ultrashort-chain and other per- and polyfluoroalkyl substances (PFAS) in wastewater treatment plants. *ACS ES T Water* 2, 1380-1390. 10.1021/acsestwater.2c00135

[DMM052320C79] Kim, J., Xin, X., Hawkins, G. L., Huang, Q. and Huang, C. H. (2024). Occurrence, fate, and removal of per- and polyfluoroalkyl substances (PFAS) in small- and large-scale municipal wastewater treatment facilities in the United States. *ACS EST Water* 4, 5428-5436. 10.1021/acsestwater.4c00541 PMC11650586 39698553

[DMM052320C80] Kleszczyński, K., Stepnowski, P. and Składanowski, A. C. (2009). Mechanism of cytotoxic action of perfluorinated acids. III. Disturbance in Ca2+ homeostasis. *Toxicol. Appl. Pharmacol.* 251, 163-168. 10.1016/j.taap.2011.01.002 21236286

[DMM052320C81] Kvalem, H. E., Nygaarda, U. C., Lødrup Carlsenb, K. C., Carlsenb, K. H., Hauga, L. S. and Granuma, B. (2020). Perfluoroalkyl substances, airways infections, allergy and asthma related health outcomes – implications of gender, exposure period and study design. *Environ. Int.* 134, 105259. 10.1016/j.envint.2019.105259 31733527

[DMM052320C82] Kwon, B. G., Lim, H. J., Na, S. H., Choi, B. I., Shin, D. S. and Chung, S. Y. (2014). Biodegradation of perfluorooctanesulfonate (PFOS) as an emerging contaminant. *Chemosphere* 109, 221-225. 10.1016/j.chemosphere.2014.01.072 24556541

[DMM052320C83] Labine, L. M., Oliveira Pereira, E. A., Kleywegt, S., Jobst, K. J., Simpson, A. J. and Simpson, M. J. (2023). Sublethal exposure of per- and polyfluoroalkyl substances of varying chain length and polar functionality results in distinct metabolic responses in daphnia magna. *Environ. Toxicol. Chem.* 42, 242-256. 10.1002/etc.5517 36345965

[DMM052320C84] Laine, M. B., Vesamäki, J. S., Puupponen, V.-M. and Tiirola, M. and Taipale S. J. (2022). Comparing the ecotoxicological effects of perfluorooctanoic acid (PFOA) and perfluorohexanoic acid (PFHxA) on freshwater microbial community. *Front. Environ. Sci.* 10, 888171. 10.3389/fenvs.2022.888171

[DMM052320C85] Levitt, D. and Liss, A. (1986). Toxicity of perfluorinated acids for human and murine B cell lines. *Toxicol. Appl. Pharmacol.* 86, 1-11. 10.1016/0041-008X(86)90394-7 3764929

[DMM052320C86] Levitt, D. and Liss, A. (1987). Perfluorinated fatty acids alter merocyanine 540 dye binding to plasma membranes. *Toxicol. Appl. Pharmacol.* 20, 303-316. 10.1080/15287398709530983 3820341

[DMM052320C87] Li K., Sun J., Yang J., Roberts S. M., Zhang X., Cui X., Wei S. and Ma L. Q. (2017). Molecular mechanisms of perfluorooctanoate-induced hepatocyte apoptosis in mice using proteomic techniques. *Environ. Sci. Technol.* 51, 11380-11389. 10.1021/acs.est.7b02690 28885018

[DMM052320C88] Li, Y., Fletcher, T., Mucs, D., Scott, K., Lindh, C. H., Tallving, P. and Jakobsson, K. (2018). Half-lives of PFOS, PFHxS and PFOA after end of exposure to conotaminated drinking water. *Occup. Environ. Med.* 75, 46-51. 10.1136/oemed-2017-104651 29133598 PMC5749314

[DMM052320C89] Li, J., Cao, J., Feng, H., Qiao, X., Zhang, A. and Fu, J. (2020a). Evaluation of the estrogenic/antiestrogenic activities of perflruoroalkyl substances and their interactions with the human estrogen receptor by combining In vitro and In silico modeling. *Environ. Sci. Technol.* 54, 14514-14524. 10.1021/acs.est.0c03468 33111528

[DMM052320C90] Li, Z., Yu, Z., Gao, P. and Yin, D. (2020b). Multigenerational effects of perfluorooctanoic acid on lipid metabolism of *Caenorhabditis elegans* and its potential mechanism. *Sci. Total Environ.* 703, 134762. 10.1016/j.scitotenv.2019.134762 31761367

[DMM052320C91] Li, F., Yu, Y., Guo, M., Lin, Y., Jiang, Y., Qu, M., Sun, X., Li, Z., Zhai, Y. and Tan, Z. (2021). Integrated analysis of physiological, transcriptomics and metabolomics provides insights into detoxication disruption of PFOA exposure in Mytilus edulis. *Ecotoxicol. Environ. Saf.* 214, 112081. 10.1016/j.ecoenv.2021.112081 33677383

[DMM052320C92] Li, J., Li, X., Da, Y., Yu, J., Long, B., Zhang, P., Bakker, C., McCarl, B. A., Yuan, J. S. and Dai, S. Y. (2022). Sustainable environmental remediation via biomimetic multifunctional lignocellulosic nano-framework. *Nat. Commun.* 13, 4368. 10.1038/s41467-022-31881-5 35902555 PMC9334262

[DMM052320C93] Lin, H., Niu, J., Xu, J., Huang, H., Li, D., Yue, Z. and Feng, C. (2013). Highly efficient and mild electrochemical mineralization of long-chain perfluorocarboxylic acids (C9-C10) by Ti/SnO2-Sb-Ce, Ti/SnO2-Sb/Ce-PbO2, and Ti/BDD electrodes. *Environ. Sci. Technol.* 47, 13039-13046. 10.1021/es4034414 24164589

[DMM052320C94] Lin, T. A., Huang, C. W. and Wei, C. C. (2022). Early-life perfluorooctanoic acid (PFOA) and perfluorooctane sulfonic acid (PFOS) exposure cause obesity by disrupting fatty acids metabolism and enhancing triglyceride synthesis in *Caenorhabditis elegans*. *Aquat. Toxicol.* 251, 106274. 10.1016/j.aquatox.2022.106274 36037606

[DMM052320C95] Liu, G., Zhang, S., Yang, K., Zhu, L. and Lin, D. (2016). Toxicity of perfluorooctane sulfonate and perfluorooctanoic acid to Escherichia coli: Membrane disruption, oxidative stress, and DNA damage induced cell inactivation and/or death. *Environ. Pollut.* 214, 806-815. 10.1016/j.envpol.2016.04.089 27155098

[DMM052320C96] Liu, Y. Z., Zhang, Z. P., Fu, Z. W., Yang, K., Ding, N., Hu, L. G., Fang, Z. Z. and Zhuo, X. (2019). Per- and polyfluoroalkyl substances display structure-dependent inhibition towards UDP-glucuronosyltransferases. *Environ. Pollut.* 254, 113093. 10.1016/j.envpol.2019.113093 31472452

[DMM052320C97] Liu, Z., Chen, Z., Gao, J., Yu, Y., Men, Y., Gu, C. and Liu, J. (2022). Accelerated degradation of perfluorosulfonates and perfluorocarboxylates by UV/Sulfite+Iodide: reaction mechanisms and system efficiencies. *Environ. Sci. Technol.* 56, 3699-3709. 10.1021/acs.est.1c07608 35226468 PMC9481055

[DMM052320C98] Liu, D., Yan, S., Wang, P., Chen, Q., Liu, Y., Cui, J., Liang, Y., Ren, S. and Gao, Y. (2023). Perfluorooctanoic acid (PFOA) exposure in relation to the kidneys: A review of current available literature. *Front. Physiol.* 14, 1103141. 10.3389/fphys.2023.1103141 36776978 PMC9909492

[DMM052320C99] Lu, S., Zhu, X., Zeng, P., Hu, L., Huang, Y., Guo, X., Chen, Q., Wang, Y., Lai, L., Xue, A. et al. (2024). Exposure of PFOA, PFOS and PFHxS induces Alzheimer's disease-like neuropathology in cerebral organoids. *Environ. Pollut.* 363, 125098. 10.1016/j.envpol.2024.125098 39389246

[DMM052320C100] Luo, Q., Lu, J., Zhang, H., Wang, Z., Feng, M., Chiang, S. Y. D., Woodward, D. and Huang, Q. (2015). Laccase catalyzed degradation of perfluorooctanoic acid. *Environ. Sci. Technol. Lett.* 2, 198-203. 10.1021/acs.estlett.5b00119

[DMM052320C101] Ma, T., Pan, X., Wang, T., Li, X. and Luo, Y. (2023). Toxicity of per- and polyfluoroalkyl substances to nematodes. *Toxics* 11, 593. 10.3390/toxics11070593 37505559 PMC10385831

[DMM052320C102] Mabaso, N. S. N., Tshangana, C. S. and Muleja, A. A. (2024). Efficient removal of PFASs using photocatalysis, membrane separation and photocatalytic membrane reactors. *Membranes* 14, 217. 10.3390/membranes14100217 39452829 PMC11509138

[DMM052320C103] Mahoney, H., Ankley, P., Roberts, C., Lamb, A., Schultz, M., Zhou, Y., Giesy, J. P. and Brinkmann, M. (2024). Unveiling the molecular effects of replacement and legacy PFASs: transcriptomic analysis of zebrafish embryos reveals surprising similarities and potencies. *Environ. Sci. Technol.* 58, 18554-18565. 10.1021/acs.est.4c04246 39392652

[DMM052320C104] Maloney, E. K. and Waxman, D. J. (1999). trans-Activation of PPARalpha and PPARgamma by structurally diverse environmental chemicals. *Toxicol. Appl. Pharmacol.* 161, 209-218. 10.1006/taap.1999.8809 10581215

[DMM052320C105] Mangu, J. C. K., Stylianou, M., Olsson, P. E. and Jass, J. (2022). Per- and polyfluoroalkyl substances enhance Staphylococcus aureus pathogenicity and impair host immune response. *Environ. Pollut.* 314, 120294. 10.1016/j.envpol.2022.120294 36181932

[DMM052320C106] Marchese, M. J., Zhu, T., Hawkey, A. B., Wang, K., Yuan, E., Wen, J., Be, S. E., Levin, E. D. and Feng, L. (2024). Prenatal and perinatal exposure to Per- and polyfluoroalkyl substances (PFAS)-contaminated drinking water impacts offspring neurobehavior and development. *Sci. Total Environ.* 917, 17045. 10.1016/j.scitotenv.2024.170459 PMC10923173 38290673

[DMM052320C107] Maxwell, D. L., Oluwaylose, O. A., Houle, E., Roth, K., Nowak, K., Sawant, S., Paskavitz, A. L., Liu, W., Gurdziel, K., Petriello, M. C. et al. (2024). Mixtures of per- and polyfluoroalkyl substances (PFAS) alter sperm methylation and long-term reprogramming of offspring liver and fat transcriptome. *Environ. Int.* 186, 108577. 10.1016/j.envint.2024.108577 38521043

[DMM052320C108] McCleaf, P., Stefansson, W. and Ahrens, L. (2023). Drinking water nanofiltration with concentrate foam fractionation—A novel approach for removal of per- and polyfluoroalkyl substances (PFAS). *Water Res.* 232, 119688. 10.1016/j.watres.2023.119688 36764110

[DMM052320C109] Merino, N., Wang, M., Ambrocio, R., Mak, K., O'Connor, E., Gao, A., Hawley, E., Deeb, R., Tseng, L. and Mahendra, S. (2018). Fungal biotransformation of 6:2 fluotelomer alcohol. *Remediat. J.* 28, 59-70. 10.1002/rem.21550

[DMM052320C110] Mirabediny, M., Sun, J., Yu, T. T., Åkermark, B., Das, B. and Kumar, N. (2023). Effective PFAS degradation by electrochemical oxidation methods-recent progress and requirement. *Chemosphere* 321, 138109. 10.1016/j.chemosphere.2023.138109 36787844

[DMM052320C111] Nassazzi, W., Wu, T. C., Jass, J., Lai, F. Y. and Ahrens, L. (2023). Phytoextraction of per- and polyfluoroalkyl substances (PFAS) and the influence of supplements on the performance of short–rotation crops. *Environ. Pollut.* 333, 122038. 10.1016/j.envpol.2023.122038 37321315

[DMM052320C112] Nassazzi, W., Bezabhe, Y. H., Guo, C., Tapase, S., Jaffe, B. D., Key, T. A., Lai, F. Y., Jass, J. and Ahrens, L. (2025). Characterization of per- and polyfluoroalkyl substances (PFAS) in willow and poplar and the impact of soil amendments on accumulation rates. *Environ. Technol. Innov.* 37, 104048. 10.1016/j.eti.2025.104048

[DMM052320C113] Oakes, K. D., Sibley, P. K., Martin, J. W., Maclean, D. D., Solomon, K. R., Mabury, S. A. and Van Der Kraak, G. J. (2005). Short-term exposures of fish to perfluorooctane sulfonate: Acute effects on fatty acyl-CoA oxidase activity, oxidative stress, and circulating sex steroids. *Environ. Toxicol. Chem.* 24, 1172-1181. 10.1897/04-419.1 16110997

[DMM052320C114] Obiako, P. C., Ayisire, S. O. and Sayes, C. M. (2024). Impact of perfluorooctanoic acid (PFOA) and perfluorobutanoic acid (PFBA) on oxidative stress and metabolic biomarkers in human neuronal cells (SH-SY5Y). *Environ. Int.* 190, 108864. 10.1016/j.envint.2024.108864 38986427

[DMM052320C115] OECD (2021). Reconciling terminology of the universe of per- and polyfluoroalkyl substances: Recommendations and practical guidance. Series on Risk Management. No. 61, OECD Publishing, Paris. 10.1787/e458e796-en

[DMM052320C116] Ojo, A. F., Xia, Q., Peng, C. and Ng, J. C. (2021). Evaluation of the individual and combined toxicity of perfluoroalkyl substances to human liver cells using biomarkers of oxidative stress. *Chemosphere* 281, 130808. 10.1016/j.chemosphere.2021.130808 34022600

[DMM052320C117] Olson, C. T. and Andersen, M. E. (1983). The acute toxicity of perfluorodecanoic acids in male rats and effects on tissue fatty acids. *Toxicol. Appl. Pharmacol.* 70, 362-372. 10.1016/0041-008X(83)90154-0 6636169

[DMM052320C118] Olsen Geary, W., Burris Jean, M., Ehresman David, J., Froehlich John, W., Seacat Andrew, M., Butenhoff John, L. and Zobel Larry R. (2007). Half-life of serum elimination of perfluorooctanesulfonate, perfluorohexanesulfonate, and perfluorooctanoate in retired fluorochemical production workers. *Environ. Health Perspect.* 115, 1298-1305. 10.1289/ehp.10009 17805419 PMC1964923

[DMM052320C119] Paul, A. G., Jones, K. C. and Sweetman, A. J. (2009). A first global production, emission, and environmental inventory for perfluorooctane sulfonate. *Environ. Sci. Technol.* 43, 386-392. 10.1021/es802216n 19238969

[DMM052320C120] Pesonen, M. and Vähäkangas, K. (2024). Involvement of per- and polyfluoroalkyl compounds in tumor development. *Arch. Toxicol.* 98, 1241-1252. 10.1007/s00204-024-03685-7 38478087 PMC10965717

[DMM052320C121] Phelps, D. W., Connors, A. M., Ferrero, G., DeWitt, J. C. and Yoder, J. A. (2024). Per- and polyfluoroalkyl substances alter innate immune function: evidence and data gaps. *J. Immunotoxicol.* 21, 2343362. 10.1080/1547691X.2024.2343362 38712868 PMC11249028

[DMM052320C122] Pizzurro, D. M., Seeley, M., Kerper, L. E. and Beck, B. D. (2019). Interspecies differences in perfluoroalkyl substances (PFAS) toxicokinetics and application to health-based criteria. *Regul. Toxicol. Pharmacol.* 106, 239-250. 10.1016/j.yrtph.2019.05.008 31078680

[DMM052320C123] Prakash, P., Randolph, C. E., Walker, K. A. and Chopra, G. (2025). Lipids: emerging players of microglial biology. *Glia* 73, 657-677. 10.1002/glia.24654 39688320 PMC11784843

[DMM052320C124] Qiao, B., Song, D., Fang, B., Yu, H., Li, X., Zhao, L., Yao, Y., Zhu, L., Chen, H. and Sun, H. (2023). Nontarget screening and fate of emerging per- and polyfluoroalkyl substances in wastewater treatment plants in Tianjin, China. *Environ. Sci. Technol.* 57, 20127-20137. 10.1021/acs.est.3c03997 37800548

[DMM052320C125] Rahman, M. F., Peldszus, S. and Anderson, W. B. (2014). Behaviour and fate of perfluoroalkyl and polyfluoroalkyl substances (PFASs) in drinking water treatment: a review. *Water Res.* 50, 318-340. 10.1016/j.watres.2013.10.045 24216232

[DMM052320C126] Rijnders, J., Bervoets, L., Prinsen, E., Eens, M., Beemster, G. T. S., AbdElgawad, H. and Groffen, T. (2021). Perfluoroalkylated acids (PFAAs) accumulate in field-exposed snails (*Cepaea sp*.) and affect their oxidative status. *Sci. Tot. Environ.* 790, 148059. 10.1016/j.scitotenv.2021.148059 34102443

[DMM052320C127] Rodríguez-Jorquera, I. A., Colli-Dula, R. C., Kroll, K., Jayasinghe, B. S., Parachu Marco, M. V., Silva-Sanchez, C., Toor, G. S. and Denslow, N. D. (2019). Blood transcriptomics analysis of fish exposed to perfluoro alkyls substances: assessment of a non-lethal sampling technique for advancing aquatic toxicology research. *Environ. Sci. Technol.* 53, 1441-1452. 10.1021/acs.est.8b03603 30572700

[DMM052320C128] Rohonczy, J., Robinson, S. A., Forbes, M. R., De Silva, A. O., Brinovcar, C., Bartlett, A. J. and Gilroy, E. A. M. (2024). The effects of two short-chain perfluoroalkyl carboxylic acids (PFCAs) on northern leopard frog (Rana pipiens) tadpole development. *Ecotoxicol* 33, 177-189. 10.1007/s10646-024-02737-z PMC10940426 38315267

[DMM052320C129] Roth, K. and Petriello, M. C. (2022). Exposure to per- and polyfluoroalkyl substances (PFAS) and type 2 diabetes risk. *Front. Endocrinol.* 13, 965384. 10.3389/fendo.2022.965384 PMC9388934 35992116

[DMM052320C130] Roth, K., Imran, Z., Liu, W. and Petriello, M. C. (2020). Diet as an exposure source and mediator of per- and polyfluoroalkyl substance (PFAS) toxicity. *Front. Toxicol.* 2, 601149. 10.3389/ftox.2020.601149 35296120 PMC8915917

[DMM052320C131] Šabović, I., Cosci, I., De Toni, L., Ferramosca, A., Stornaiuolo, M., Di Nisio, A., Dall'Acqua, S., Garolla, A. and Foresta, C. (2020). Perfluoro-octanoic acid impairs sperm motility through the alteration of plasma membrane. *J. Endocrinol. Invest.* 43, 641-652. 10.1007/s40618-019-01152-0 31776969

[DMM052320C133] Sana, T., Chowdhury, M. I., Logeshwaran, P., Dharmarajan, R. and Megharaj, M. (2021). Perfluorooctanoic acid (PFOA) induces behavioural, reproductive and developmental toxicological impacts in *Caenorhabditis elegans* at concentrations relevant to the contaminated areas. *Environ. Adv.* 4, 100053. 10.1016/j.envadv.2021.100053

[DMM052320C134] Sana, T., Chowdhury, M. I., Logeshwaran, P. and Megharaj, M. (2023). Behavioural, developmental and reproductive toxicological impacts of perfluorobutanoic acid (PFBA) in *Caenorhabditis elegans*. *Environ. Chall.* 10, 100662. 10.1016/j.envc.2022.100662

[DMM052320C135] Schaefer, C. E., Hooper, J. L., Strom, L. E., Abusallout, I., Dickenson, E. R. V., Thompson, K. A., Mohan, G. R., Drennan, D., Wu, K. and Guelfo, J. L. (2023). Occurrence of quantifiable and semi-quantifiable poly- and perfluoroalkyl substances in united states wastewater treatment plants. *Water Res.* 233, 119724. 10.1016/j.watres.2023.119724 36801573

[DMM052320C136] Seyoum, A., Pradhan, A., Jass, J. and Olsson, P. E. (2020). Perfluorinated alkyl substances impede growth, reproduction, lipid metabolism and lifespan in *Daphnia magna*. *Sci. Tot. Environ.* 737, 139682. 10.1016/j.scitotenv.2020.139682 32521362

[DMM052320C137] Shahsavari, E., Rouch, D., Khudur, L. S., Thomas, D., Aburto-Medina, A. and Ball, A. S. (2021). Challenges and current status of the biological treatment of PFAS-contaminated soils. *Front. Bioeng. Biotechnol.* 8, 602040. 10.3389/fbioe.2020.602040 33490051 PMC7817812

[DMM052320C138] Shang, Y., Chen, K., Ni, H., Zhu, X., Yuan, X., Wang, Y., Liu, X., Cui, Z., Niu, Y., Shi, Y. et al. (2024). Environmentally relevant concentrations of perfluorobutane sulfonate impair locomotion behaviors and healthspan by downregulating mitophagy in *C. elegans*. *J. Hazard. Mater.* 480, 135938. 10.1016/j.jhazmat.2024.135938 39326150

[DMM052320C139] Shao, X., Ji, F., Wang, Y., Zhu, L., Zhang, Z., Du, X., Chung, A. C. K., Hong, Y., Zhao, Q. and Cai, Z. (2018). Integrative chemical proteomics-metabolomics approach reveals Acaca/Acacb as direct molecular targets of PFOA. *Anal. Chem.* 90, 11092-11098. 10.1021/acs.analchem.8b02995 30134650

[DMM052320C140] Shaw, D. M. J., Munoz, G., Bottos, E. M., Duy, S. V., Sauvé, S., Jinxia, L. and Van Hamme, J. D. (2019). Degradation and defluorination of 6:2 fluorotelomer sulfonamidoalkyl betaine and 6:2 fluorotelomer sulfonate by Gordonia sp. strain NB4-1Y under sulfur-limiting conditions. *Sci. Total Environ.* 647, 690-698. 10.1016/j.scitotenv.2018.08.012 30092525

[DMM052320C141] Shelly, C., Grandjean, P., Oulhote, Y., Plomgaard, P., Frikke-Schmidt, R., Nielsen, F., Zmirou-Navier, D., Weihe, P. and Valvi, D. (2019). Early life exposures to perfluoroalkyl substances in relation to adipokine hormone levels at birth and during childhood. *J Clin. Endocrinol. Metab.* 104, 5338-5348. 10.1210/jc.2019-00385 31216000 PMC6773461

[DMM052320C142] Shi, G., Guo, H., Sheng, N., Cui, Q., Pan, Y., Wang, J., Guo, Y. and Dai, J. (2018). Two-generational reproductive toxicity assessment of 6:2 chlorinated polyfluorinated ether sulfonate (F-53B, a novel alternative to perfluorooctane sulfonate) in zebrafish. *Environ. Pollut.* 243, 1517-1527. 10.1016/j.envpol.2018.09.120 30292160

[DMM052320C143] Shipley, J. M., Hurest, C. H., Tanaka, S. S., DeRoos, F. L., Butenhoff, J. L., Seacat, A. M. and Waxman, D. J. (2004). Trans-activation of PPARalpha and induction of PPARalpha target genes by perfluorooctane-based chemicals. *Toxicol. Sci.* 80, 151-160. 10.1093/toxsci/kfh130 15071170

[DMM052320C144] Singh, N. and Hsieh, C. Y. J. (2021). Exploring potential carcinogenic activity of per- and polyfluorinated Alkyl substances utilizing high-throughput toxicity screening data. *Int. J. Toxicol.* 40, 355-366. 10.1177/10915818211010490 33944624

[DMM052320C145] Smith, S. J., Lauria, M., Ahrens, L., McCleaf, P., Hollman, P., Bjälkefur Seroka, S., Hamers, T., Arp, H. P. H. and Wiberg, K. (2023). Electrochemical oxidation for treatment of PFAS in contaminated water and fractionated foam – A pilot-scale study. *ACS ES T Water* 3, 1201-1211. 10.1021/acsestwater.2c00660 37090120 PMC10111409

[DMM052320C146] Sonnino, S., Aureli, M., Mauri, L., Ciampa, M. G. and Prinetti, A. (2015). Membrane lipid domains in the nervous system. *Front Biosci (Landmark Ed)* 20, 280-302. 10.2741/4309 25553451

[DMM052320C147] Sorn, S., Hara-Yamamura, H., Vet, S., Xiao, M., Hoek, E. M. V. and Honda, R. (2023). Biological treatment of perfluorooctanesulfonic acid (PFOS) using microbial capsules of a polysulfone membrane. *Chemosphere* 329, 138585. 10.1016/j.chemosphere.2023.138585 37028728

[DMM052320C148] Sprengel, J., Behnisch, P. A., Besselink, H., Bouwer, A. and Vetter, W. (2021). In vitro human cell-based TTR-TRβ CALUX assay indicates thyroid hormone transport disruption of short-chain, medium-chain and long-chain chlorinated paraffins. *Arch. Toxicol.* 95, 1391-1396. 10.1007/s00204-021-02994-5 33555371 PMC8032603

[DMM052320C149] Stein, C. R., Sacitz, D. A. and Dougan, M. (2009). Serum levels of perfluorooctanoic acid and perfluorooctane sulfonate and pregnancy outcome. *Am. J. Epidemiol.* 170, 837-846. 10.1093/aje/kwp212 19692329

[DMM052320C150] Stein, C. R., McGovern, K. J., Pajak, A. M., Maglione, P. J. and Wolff, M. S. (2015). Perfluoroalkyl and polyfluoroalkyl substances and indicators of immune function in children aged 12-19 y: national health and nutrition examination survey. *Pediatr. Res.* 79, 348-357. 10.1038/pr.2015.213 26492286 PMC5065061

[DMM052320C151] Stylianou, M., Björnsdotter, M. K., Olsson, P.-E., Ericson Jogsten, I. and Jass, J. (2019). Distinct transcriptional response of *Caenorhabditis elegans* to different exposure routes of perfluorooctane sulfonic acid. *Envir. Res.* 168, 406-413. 10.1016/j.envres.2018.10.019 30388497

[DMM052320C152] Sudharshan, S. J., Tirupathi, R. and Dyavaiah, M. (2019). Astaxanthin reduces perfluorooctanoic acid cytotoxicity in Saccharomyces cerevisiae. *Toxicol. Res.* 8, 1009-1015. 10.1039/c9tx00215d PMC7478099 32922741

[DMM052320C154] Taher, M. N., Al-Mutwalli, S. A., Barisci, S., Koseoglu-Imer, D. Y., Dumée, L. F. and Shirazi, M. M. A. (2024). Progress on remediation of per- and polyfluoroalkyl substances (PFAS) from water and wastewater using membrane technologies: a review. *J. Water Process. Eng.* 59, 104858. 10.1016/j.jwpe.2024.104858

[DMM052320C155] Taves, D. (1968a). Evidence that there are two forms of ﬂuorine in human serum. *Nature* 217, 1050-1051. 10.1038/2171050b0 4171201

[DMM052320C156] Taves, D. (1968b). Electrophoretic mobility of serumﬂuoride. *Nature* 220, 582-583. 10.1038/220582a0 5686731

[DMM052320C157] Taves, D., Guy, W. and Brey, W. (1976). Organic ﬂuoro-carbons in human plasma: prevalence and characterization. In *Biochemistry Involving Carbon-ﬂuorine Bonds* (ed. R. Filler), pp. 117-134. Washington, DC: American Chemical Society.

[DMM052320C158] Taylor, M. D., Bräunig, J., Mueller, J. F., Crompton, M., Dunstan, R. H. and Nilsson, S. (2019). Metabolomic profiles associated with exposure to per- and polyfluoroalkyl substances (PFASs) in aquatic environments. *Environ. Sci.* 21, 1980-1990. 10.1039/C9EM00394K 31553340

[DMM052320C160] Timmermann, C. A. G., Jensen, K. J., Nielsen, F., Budtz-Jørgensen, E., van der Klis, F., Benn, C. S., Grandjean, P. and Fisker, A. B. (2020). Serum perfluoroalkyl substances, vaccine responses, and morbidity in a cohort of guinea-bissau children. *Environ. Health Perspect.* 128, 87002. 10.1289/EHP6517 32772733 PMC7416537

[DMM052320C161] Torres-Farradá, G., Thijs, S., Rineau, F., Guerra, G. and Vangronsveld, J. (2024). White rot fungi as tools for the bioremediation of xenobiotics: a review. *J. Fungi* 10, 167. 10.3390/jof10030167 PMC10971306 38535176

[DMM052320C162] Tseng, N., Wang, N., Szostek, B. and Mahendra, S. (2014). Biotransformation of 6:2 fluorotelomer alcohol (6:2 FTOH) by a wood-rotting fungus. *Environ. Sci. Technol.* 48, 4012-4020. 10.1021/es4057483 24593855

[DMM052320C163] Tursi, Q. R., Lindeman, B., Kristoffersen, A. B., Hjertholm, H., Bronder, E., Andreassen, M., Husøy, T., Dirven, H., Andorf, S. and Nygaard, U. C. (2024). Immune cell profiles associated with human exposure to perfluorinated compounds (PFAS) suggest changes in natural killer, T helper, and T cytotoxic cell subpopulations. *Environ. Res.* 256, 119221. 10.1016/j.envres.2024.119221 38795951 PMC11934339

[DMM052320C164] Urtiaga, A. (2021). Electrochemical technologies combined with membrane filtration. *Curr. Opin. Electrochem.* 27, 100691. 10.1016/j.coelec.2021.100691

[DMM052320C165] Uwayezu, J. N., Carabante, I., van Hees, P., Karlsson, P. and Kumpiene, J. (2023). Validation of UV/persulfate as a PFAS treatment of industrial wastewater and environmental samples. *J. Water Process. Eng.* 53, 103614. 10.1016/j.jwpe.2023.103614

[DMM052320C166] Villeneuve, D. L., Blackwell, B. R., Bush, K., Harrill, J., Harris, F., Hazemi, M., Le, M., Stacy, E. and Flynn, K. M. (2024). Transcriptomics-based points of departure for daphnia magna exposed to 18 Per- and Polyfluoroalkyl substances. *Environ. Toxicol. Chem.* 44, 2470-2484. 10.1002/etc.5838 38450772

[DMM052320C167] von Holst, H., Nayak, P., Dembek, Z., Buehler, S., Echeverria, D., Fallacara, D. and John, L. (2021). Perfluoroalkyl substances exposure and immunity, allergic response, infection, and asthma in children: review of epidemiologic studies. *Heliyon* 7, e08160. 10.1016/j.heliyon.2021.e08160 34712855 PMC8529509

[DMM052320C168] Wasel, O., Thompson, K. M., Gao, Y., Godfrey, A. E., Gao, J., Mahapatra, C. T., Lee, L. S., Sepulveda, M. S. and Freeman, J. L. (2021). Comparison of zebrafish in vitro and in vivo developmental toxicity assessments of perfluoroalkyl acids (PFAAs). *J. Toxicol. Environ. Health A* 84, 125-136. 10.1080/15287394.2020.1842272 33143551

[DMM052320C169] Wang, T., Wang, Y., Liao, C. and Jiang, G. (2009). Perspectives on the inclusion of perfluorooctane sulfonate into the Stockholm convention on persistent organic pollutants. *Environ. Sci. Technol.* 43, 5171-5175. 10.1021/es900464a 19708337

[DMM052320C170] Wang, Z., Cousins, I. T., Scheringer, M. and Hungerbuhler, K. (2013). Fluorinated alternatives to long-chain perfluoroalkyl carboxylic acids (PFCAs), perfluoroalkane sulfonic acids (PFSAs) and their potential precursors. *Environ. Int.* 60, 242-248. 10.1016/j.envint.2013.08.021 24660230

[DMM052320C171] Wang, Z., Buser, A. M., Cousins, I. T., Demattio, S., Drost, W., Johansson, O., Ohno, K., Patlewicz, G., Richared, A. M., Walker, G. W. et al. (2021). A new definition for per- and polyfluoroalkyl substances. *Environ. Sci. Technol.* 55, 155575-155578. 10.1021/acs.est.1c06896 34751569

[DMM052320C172] Wang, Q., Ruan, Y., Jin, L., Tao, L. S. R., Lai, H., Li, G., Yeung, W. Y., Leung, K. M. Y. and Lam, P. K. S. (2023). Legacy and emerging Per- and Polyfluoroalkyl substances in a subtropical marine food web: suspect screening, isomer profile, and identification of analytical interference. *Environ. Sci. Technol.* 57, 8355-8364. 10.1021/acs.est.3c00374 37220884 PMC10249352

[DMM052320C173] Wang, L., Yang, T., Liu, X., Liu, J. and Liu, W. (2024a). Critical evaluation and meta-analysis of ecotoxicological data on per- and polyfluoroalkyl substances (PFAS) in freshwater species. *Environ. Sci. Technol.* 58, 17555-17566. 10.1021/acs.est.4c04818 39316471

[DMM052320C174] Wang, Q., Ruan, Y., Shao, Y., Jin, L., Xie, N., Yan, M., Chen, L., Schlenk, D., Leung, K. M. Y. and Lam, P. K. S. (2024b). Stereoselective bioconcentration and neurotoxicity of perfluoroethylcyclohexane sulfonate in marine Medaka. *Environ. Sci. Technol.* 58, 12933-12942. 10.1021/acs.est.4c03571 39003765

[DMM052320C175] Wee, S. Y. and Aris, A. Z. (2023). Revisiting the “forever chemicals”, PFOA and PFOS exposure in drinking water. *NPJ Clean Water* 6, 57. 10.1038/s41545-023-00274-6

[DMM052320C176] Wei, C., Zhou, Z., Wang, L., Huang, Z., Liang, Y. and Zhang, J. (2021). Perfluorooctane sulfonate (PFOS) disturbs fatty acid metabolism in *Caenorhabditis elegans*: evidence from chemical analysis and molecular mechanism exploration. *Chemosphere* 277, 130359. 10.1016/j.chemosphere.2021.130359 34384190

[DMM052320C177] Wu, S., Xie, J., Zhao, H., Sanchez, O. F., Rochet, J. C., Freeman, J. L. and Yuan, C. (2024). Developmental neurotoxicity of PFOA exposure on hiPSC-derived cortical neurons. *Environ. Int.* 190, 108914. 10.1016/j.envint.2024.108914 39079332 PMC11406754

[DMM052320C178] Xu, Y., Lindh, C. H., Fletcher, T., Jakobsson, K. and Engström, K. (2022). Perfluoroalkyl substances influence DNA methylation in school-age children highly exposed through drinking water contaminated from firefighting foam: a cohort study in Ronneby, Sweden. *Environ. Epigenet.* 8, dvac004. 10.1093/eep/dvac004 35308102 PMC8931254

[DMM052320C179] Yan, X. Z., Peng, J., Liu, Y. Q., Fan, R. N., Ni, X. Y., Gong, L., Zhang, D. N., Huang, X., Tan, S. H. and Wang, H. L. (2025). Mixed exposure to PFOA and PFOS induces oocyte apoptosis and subfertility in mice by activating the Hippo signaling pathway. *Reprod. Toxicol.* 132, 108829. 10.1016/j.reprotox.2024.108829 39746460

[DMM052320C188] Yang, H.B., Zhao, Y.Z., Tang, Y., Gong, H.Q., Guo, F., Sun, W.H., Liu, S.S., Tan, H. and Chen, F. (2019). Antioxidant defence system is responsible for the toxicological interacions of mixtures: A case study of PFOS and PFOA in Daphnia magna. *Sci. Totla Environ.* 667, 435-443. 10.1016/j.scitotenv.2019.02.418 30833242

[DMM052320C180] Yuan, Z., Shao, X., Miao, Z., Zhao, B., Zheng, Z. and Zhang, J. (2018). Perfluorooctane sulfonate induced neurotoxicity responses associated with neural genes expression, neurotransmitter levels and acetylcholinesterase activity in planarians *Dugesia japonica*. *Chemosphere* 206, 150-156. 10.1016/j.chemosphere.2018.05.011 29738904

[DMM052320C181] Zhang, H., He, J., Li, N., Gao, N., Du, Q., Chen, B., Chen, F., Shan, X., Ding, Y., Zhu, W. et al. (2019). Lipid accumulation responses in the liver of Rana nigromaculata induced by perfluorooctanoic acid (PFOA). *Ecotoxicol. Environ. Saf.* 167, 29-35. 10.1016/j.ecoenv.2018.09.120 30292973

[DMM052320C182] Zhang, L., Sun, W., Chen, H., Tian, F. and Cai, W. (2020). Transcriptome analysis of acute exposure of the Manila clam, Ruditapes philippinarum to perfluorooctane sulfonate (PFOS). *Comp. Biochem. Physiol. C Toxicol. Pharmacol.* 231, 108736. 10.1016/j.cbpc.2020.108736 32142923

[DMM052320C183] Zhang, Y., Mustieles, V., Sun, Y., Oulhote, Y., Wang, Y. X. and Messerlian, C. (2023). Association between serum per- and polyfluoroalkyl substances concentrations and common cold among children and adolescents in the United States. *Environ. Int.* 164, 107239. 10.1016/j.envint.2022.107239 PMC10250187 35447424

[DMM052320C184] Zhang, Y., Zhou, Y., Dong, R., Song, N., Hong, M., Li, J., Yu, J. and Kong, D. (2024). Emerging and legacy per- and polyfluoroalkyl substances (PFAS) in fluorochemical wastewater along full-scale treatment processes: source, fate, and ecological risk. *J. Hazard Mater.* 465, 133270. 10.1016/j.jhazmat.2023.133270 38113743

[DMM052320C185] Zhao, S., Wang, B., Zhong, Z., Liu, T., Liang, T. and Zhan, J. (2020). Contributions of enzymes and gut microbes to biotransformation of perfluorooctane sulfonamide in earthworms (Eisenia fetida). *Chemosphere* 238, 124619. 10.1016/j.chemosphere.2019.124619 31450114

[DMM052320C186] Zhao, L., Teng, M., Zhao, X., Li, Y., Sun, J., Zhao, W., Ruan, Y., Leung, K. M. Y. and Wu, F. (2023). Insight into the binding model of polyfluoroalkyl substances to proteins and membranes. *Environ. Int.* 175, 107951. 10.1016/j.envint.2023.107951 37126916

